# Green synthesis of bifunctional phthalocyanine-porphyrin COFs in water for efficient electrocatalytic CO_2_ reduction coupled with methanol oxidation

**DOI:** 10.1093/nsr/nwad226

**Published:** 2023-09-02

**Authors:** Mi Zhang, Jia-Peng Liao, Run-Han Li, Sheng-Nan Sun, Meng Lu, Long-Zhang Dong, Pei Huang, Shun-Li Li, Yue-Peng Cai, Ya-Qian Lan

**Affiliations:** School of Chemistry, South China Normal University, Guangzhou 510006, China; School of Chemistry, South China Normal University, Guangzhou 510006, China; School of Chemistry, South China Normal University, Guangzhou 510006, China; School of Chemistry, South China Normal University, Guangzhou 510006, China; School of Chemistry, South China Normal University, Guangzhou 510006, China; School of Chemistry, South China Normal University, Guangzhou 510006, China; School of Chemistry, South China Normal University, Guangzhou 510006, China; School of Chemistry, South China Normal University, Guangzhou 510006, China; School of Chemistry, South China Normal University, Guangzhou 510006, China; School of Chemistry, South China Normal University, Guangzhou 510006, China

**Keywords:** hydrothermal synthesis, phthalocyanine-porphyrin COFs, bifunctional electrocatalysts, electrocatalytic CO_2_ reduction, methanol oxidation reaction

## Abstract

Electrocatalytic CO_2_ reduction (ECR) coupled with organic oxidation is a promising strategy to produce high value-added chemicals and improve energy efficiency. However, achieving the efficient redox coupling reaction is still challenging due to the lack of suitable electrocatalysts. Herein, we designed two bifunctional polyimides-linked covalent organic frameworks (PI-COFs) through assembling phthalocyanine (Pc) and porphyrin (Por) by non-toxic hydrothermal methods in pure water to realize the above catalytic reactions. Due to the high conductivity and well-defined active sites with different chemical environments, NiPc-NiPor COF performs efficient ECR coupled with methanol oxidation reaction (MOR) (Faradaic efficiency of CO (FE_CO_) = 98.12%, partial current densities of CO (j_CO_) = 6.14 mA cm^−2^ for ECR, FE_HCOOH_ = 93.75%, j_HCOOH_ = 5.81 mA cm^−2^ for MOR at low cell voltage (2.1 V) and remarkable long-term stability). Furthermore, experimental evidences and density functional theory (DFT) calculations demonstrate that the ECR process mainly conducts on NiPc unit with the assistance of NiPor, meanwhile, the MOR prefers NiPor conjugating with NiPc. The two units of NiPc-NiPor COF collaboratively promote the coupled oxidation-reduction reaction. For the first time, this work achieves the rational design of bifunctional COFs for coupled heterogeneous catalysis, which opens a new area for crystalline material catalysts.

## Teaser

Bifunctional phthalocyanine-porphyrin COFs were synthesized by hydrothermal method without using catalyst/toxic solvent, and simultaneously possessed high crystallinity, conductivity and multiple active-sites, which exhibited high activity for CO_2_ electro-reduction coupled with methanol electro-oxidation.

## INTRODUCTION

The massive consumption of fossil fuels all over the world has led to excessive CO_2_ emissions into the atmosphere, which has caused serious environmental issues and energy crises [1–3]. Electrochemical CO_2_ reduction (ECR) by renewable electric energy offers a promising strategy to convert CO_2_ into useful energy substances, such as CO, CH_3_OH, HCOOH, CH_4_ and C_2_H_4_  *et al*., which will simultaneously reduce CO_2_ and produce useful energy fuels [4–8]. In recent years, multifarious ECR catalysts have been developed for CO_2_ reduction, and high efficiencies have also been achieved, which showed great future prospects for practical uses. However, most studies only focused on the ECR half reaction at the cathode when evaluating catalytic performance, while neglecting the relevant oxidation half reaction on the anode side, thus causing a great waste of energy [9,10]. In most of the reported researches, the conventional method to treat anode reaction has been coupled with the water oxidation reaction (oxygen evolution reaction, OER) by using a carbon or platinum rod as the anode [11,12]. Unfortunately, this OER process will cause overpotential and also needs a high energy input due to slow kinetics and unfavorable thermodynamics of H_2_O oxidation reaction, thus leading to lower energy efficiency for the overall catalytic reaction. Besides, the O_2_ produced is relatively less value-added compared to many industrial chemicals [13,14]. Therefore, there is an urgent need to develop an oxidation reaction with high energy efficiency to replace the OER process.

The application of the anodic oxidation process to the organic molecules oxidative synthesis, such as methanol oxidation reaction (MOR) to produce HCOOH, can effectively improve energy efficiency due to low theoretical overpotential and also be in line with the demand of green chemistry [15–17]. However, it remains a challenge to enable these two electrocatalytic reactions to cooperate effectively. The main barrier in this field is the lack of highly active multifunctional electrocatalysts to fulfill these two processes. Theoretically, the electrocatalysts for ECR coupled with MOR should satisfy the following requirements: (1) highly active and accessible catalytic sites for reduction or oxidation reaction [18]; (2) affinity and adsorption activation for substrates such as CO_2_ or methanol [19]; (3) preferable electron and proton transfer ability [20]; (4) high stability during the electrochemical measurements [21]. Until now, many researches have explored the activity of a single functional homogeneous catalyst (such as metal complex) for ECR or MOR separately, while the problems of recycling and stability are still difficult to solve [22,23]. The construction of bifunctional heterogeneous catalysts can effectively solve the above problems which is used for ECR coupled with MOR, yet this has rarely been studied. Among them, the well-defined model with precise structure is particularly important for studying the structure-function relationship and mechanism of bifunctional heterogeneous catalysts.

**Scheme 1.**
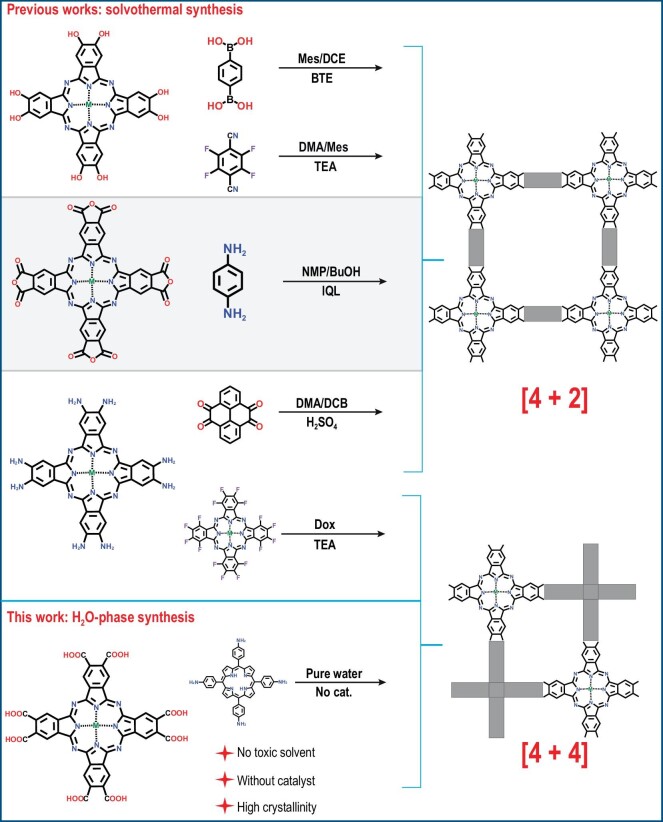
Schematic representation of design and synthesis Pc-based COFs. This work and previous works. (Mesitylene (Mes), 1,2-dichloroethane (DCE), Boron trifluoride etherate (BTE), N, N-Dimethylacetamide (DMA), Triethylamine (TEA), N-methylpyrrolidone (NMP), 1-butanol (BuOH), Isoquinoline (IQL), 1,2-dichlorobenzene (DCB), Sulfuric acid (H_2_SO_4_), 1,4-Dioxane (Dox)).

Covalent organic frameworks (COFs) with excellent structural designability and high stabilities are promising platforms for catalytic reactions [24–27]. Some building blocks of COFs possess appropriate coordination sites, thus making them capable of introducing metal active sites for typical catalysis [28,29]. Up to the present, COFs-based catalysts have been successfully applied for ECR, OER and oxygen reduction reaction (ORR), *et al*., which illustrates the great potential for electrocatalysis [30,31]. However, the precise introduction of multiple active sites with different chemical environments into COFs is still in its infancy, much less their application in electrocatalysis. Recently, metallophthalocyanine (MPc) and metalloporphyrin (MPor) based-COFs have been studied for catalytic reactions [32,33]. Nevertheless, most of these works only focused on studying the catalytic performance of a single functional component, while the integration of MPc and MPor together into crystalline COFs for bifunctional catalysts was still unexplored. Besides, as one of the most important classes of crystalline COFs, Pc-based COFs possess excellent conductivity, mechanical performance and redox-active properties [34,35]. However, the traditional synthesis of Pc-based COFs based on solvothermal methods will inevitably use toxic organic solvents and catalysts [36–40]. Therefore, it is necessary to develop green and efficient methods to synthesize crystalline Pc-based COFs (Scheme 1). Unterlass *et al.* demonstrated that highly crystalline all-aromatic polyimides can be synthesized by hydrothermal polymerization using only H_2_O as solvent [41]. Besides, the alcohol-assisted hydrothermal synthesis which was developed by Lotsch *et al.* also confirmed imide-linkage can be obtained without a toxic solvent [42]. On the basis of the above research results, we successfully get a series of crystalline Pc-based COFs. It is noted that our report is the first synthesis of highly crystalline Pc-based COFs by hydrothermal synthesis in pure water without using catalyst and toxic solvents, which conforms to green synthesis chemistry. According to the above H_2_O-phase synthesis method, we rationally prepared crystalline NiPc-2HPor COF by condensing a phthalic acid group of NiPc and aromatic amine group of 2HPor through hydrothermal methods (Scheme 1), and further synthesized NiPc-NiPor COF by post-synthesis coordination reaction. The polyimides-linked COFs (PI-COFs) which were formed showed high chemical stability and activity for electrocatalysis MOR coupled with ECR. The porous NiPc-NiPor COF structure not only plays the role of metal site supports, but also possesses high conductivity and regularity. Besides, the Ni in the pockets of MPc and MPor with different chemical environments can act as synergy active sites, thus greatly enhancing ECR coupling MOR catalytic performance.

Above all, the synthesized NiPc-MPor COFs combining the features of crystallinity and conductivity, also have multiple active sites with different chemical environments for ECR and MOR. Among them, the NiPc-NiPor COF shows excellent activity for cathodic ECR (FE_CO_ = 98.12%, j_CO_ = 6.14 mA cm^−2^) coupled with anodic MOR to HCOOH (FE_HCOOH_ = 93.75%, j_HCOOH_ = 5.81 mA cm^−2^) in a H-cell at low cell voltage (2.1 V) and exhibits remarkable long-term stability, which is comparable to most reported ECR-MOR coupled catalysts. The *in-situ* Fourier transform infrared spectroscopy (FT-IR) was used to identify the key intermediates for both ECR and MOR. Furthermore, the density functional theory (DFT) calculations demonstrate that the ECR process mainly performs on NiPc unit with the assistance of NiPor, meanwhile, the MOR process shows a preference for NiPor and conjugates with NiPc. The synergistic catalytic effect of NiPc and NiPor combined contributes to such high catalytic activity. This is the first report of bifunctional MPc-MPor-based COFs for electrocatalytic cathodic ECR coupled with anodic MOR simultaneously, which is also of great significance in the field of bifunctional electrocatalysts.

## RESULTS AND DISCUSSION

### Synthesis and structure of NiPc-MPor COF

**Figure 1.**
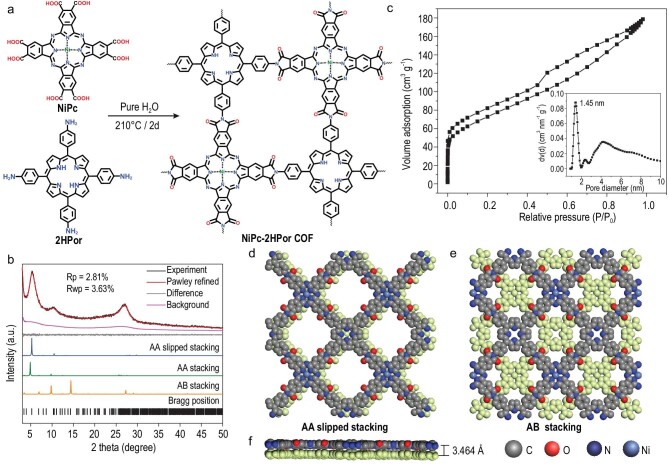
Schematic representation of metallophthalocyanine-porphyrin COFs by polyimide linkage. (a) Schematic of the synthesis and structure of NiPc-2HPor COF through the condensation of NiPc and 2HPor. (b) Simulated and experiment PXRD patterns of NiPc-2HPor COF. (c) N_2_ adsorption isotherm of NiPc-2HPor COF. Inset is the pore size distribution. (d) Crystal structure of simulated AA slipped stacking and (e) AB stacking for NiPc-2HPor COF. (f) Side view of AA slipped stacking mode for NiPc-2HPor COF.

**Figure 2.**
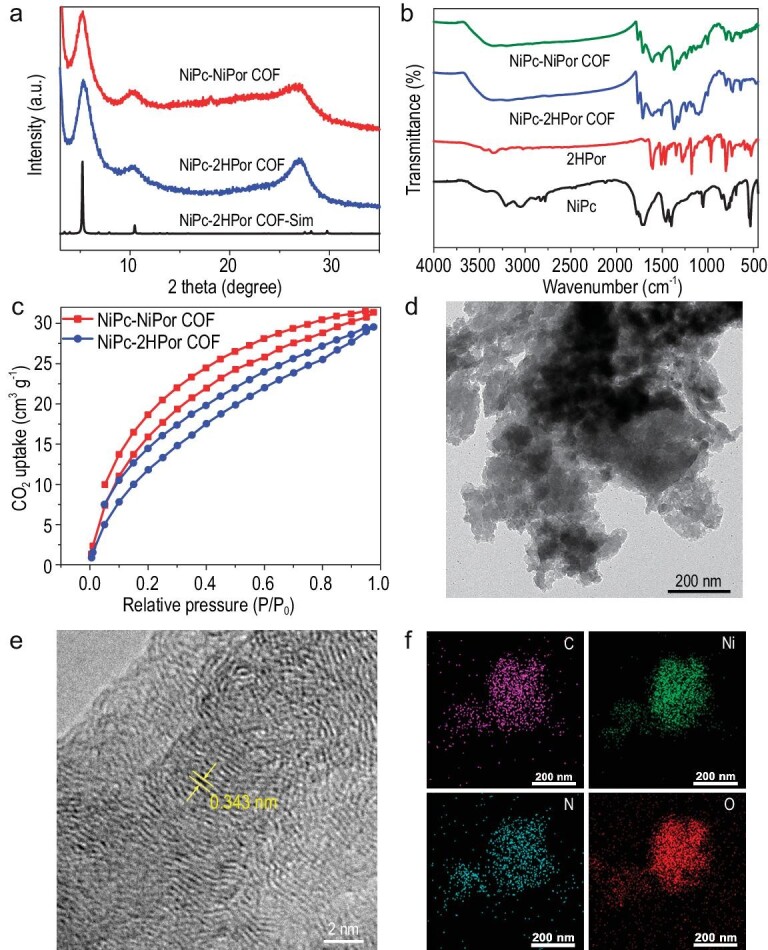
Characterization of NiPc-MPor COFs. (a) The PXRD curve of NiPc-MPor COFs. (b) FT-IR of NiPc-MPor COFs. (c) CO_2_ adsorption-desorption isotherms of NiPc-MPor COFs. (d) TEM image of NiPc-NiPor COF. (e) HRTEM image of NiPc-NiPor COF. (f) Element mapping of NiPc-NiPor COF.

As shown in Scheme 1 and Fig. 1, a [4 + 4] condensation reaction is applied to synthesize NiPc-2HPor COF. Specifically, NiPc-2HPor COF was synthesized by condensation between 2,3,9,10,16,17,23,24-octacarboxyphthalocyanine nickel (NiPc) and 5,10,15,20-tetrakis(para-aminophenyl) porphyrin (2HPor) via hydrothermal methods (Fig. 1a). The crystal structure of NiPc-2HPor COF was characterized by powder X-ray diffraction (PXRD) measurements combined with structural simulation. The AA and AB stacking structural model was constructed based on reticular chemistry, while the results showed that the theoretical PXRD patterns of AA and AB model had some deviations from the experimental curves (Fig. 1b). Interestingly, we found that the theoretical PXRD pattern of AA slipped stacking with *Pm* (6) space group model fitted well with the experimental one (for details, see the structural modeling section). Therefore, we then conducted Pawley refinement based on AA slipped stacking against the experimental PXRD pattern, which provided unit cell parameters of a = b = 25.7859 Å, c = 3.4637 Å, α = γ = 90°, β = 120°. The refinement of PXRD diffraction patterns fitted well with the experimental results, with residuals of Rp = 2.81% and Rwp = 3.63%, thus confirming the accuracy of the simulated structure. The peaks at 5.27° and 10.54° are assigned to the (110) and (220) planes, respectively. The porosity of NiPc-2HPor COF was then determined by N_2_ adsorption isotherms at 77 K, and the results showed that the pore size distributed at 1.45 nm, which was consistent with the theoretical aperture (Fig. 1c–f). The BET surface area of NiPc-2HPor COF was calculated to be 258.608 m^2^ g^−1^. Based on the NiPc-2HPor COF, we further synthesized NiPc-NiPor COF by post-synthesis coordination reaction (Scheme S2). The synthesized NiPc-NiPor COF also shows high crystallinity as confirmed by the PXRD pattern (Fig. 2a). The comparison of PXRD patterns of NiPc-2HPor COF and NiPc-NiPor COF with 2HPor and NiPc show no precursor monomers exist, suggesting the completeness of the polymerization reaction (Fig. S1). Fourier transform infrared (FT-IR) was then conducted to characterize the chemical structure which confirmed the imide formation reaction in NiPc-2HPor COF and NiPc-NiPor COF. As shown in Fig. 2b, the obvious peaks at 1762 and 1707 cm^−1^ correspond to asymmetric and symmetric vibrations of C=O of the five-membered imide rings and the peaks at 1368 and 1324 cm^−1^ belong to the stretching vibration of the C-N-C bond of polyimide [43]. Furthermore, the peaks corresponding to the carboxylic acid of the precursor NiPc at 1696 cm^−1^ and the amide bond of the NiPor at 1674 cm^−1^ are not observed, which indicates full imidization yielding the desired PI-COFs (Fig. 2b). The thermostability of COFs was studied by thermogravimetric analysis (TGA) under N_2_ and O_2_ atmosphere (Figs S2–S5), which showed no obvious change up to ∼300^o^C under both nitrogen and oxygen atmospheres. X-ray photoelectron spectroscopy (XPS) was conducted to confirm all the element states over COFs (Figs S6–S10), which showed C, N, O and Ni coexisting in NiPc-2HPor COF and NiPc-NiPor COF. Furthermore, the analysis results show the divalent state of the central Ni in COFs. We then performed high-resolution XPS and their deconvolution for C1s, N1s and O1s. In the high-resolution N1s spectrum of NiPc-2HPor COF (Fig. S10a), the binding energy peaks at 398.8, 399.2, 399.8 and 400.5 eV corresponding to iminic N, C = N, pyrrolic N and C-N, respectively. As a result, the disappearance of the pyrrolic N peak in NiPc-NiPor COF (Fig. S10d and Table S1) shows the successful post-metalation of Ni [44].

All above results illustrate the successful condensation of NiPc and 2HPor and the formation of PI-COFs. We then performed the CO_2_ adsorption-desorption test of these COFs. As shown in Fig. 2c, NiPc-NiPor COF have a strong CO_2_ adsorption capacity of about 31.19 cm^3^ g^−1^ in 273 K, which is higher than the NiPc-2HPor COF, and thus is more beneficial for the ECR reaction. The crystal morphology of these COFs was observed by transmission electron microscopy (TEM) and scanning electron microscopy (SEM). The TEM shows that NiPc-NiPor/2HPor COF displays a lamellar shape crystal with a size of ∼50–100 nm (Fig. 2d and Fig. S11). The SEM images of COFs further confirm the microcrystal morphology (Figs S12 and S13). Furthermore, the high-resolution TEM (HRTEM) of NiPc-NiPor COF displays clear lattice fringes of (001) crystal face with a distance of 0.343 nm, which fits well with the theoretical layer distance (0.346 nm), further confirming the precise nature of the simulated crystal structure (Figs 1f and 2e). Energy dispersive X-ray spectroscopy (EDX) mapping reveals the uniform distribution of the Ni, C, N and O element of NiPc-NiPor COF, which illustrates the homogeneity of these materials (Fig. 2f).

### Electrocatalytic ECR coupling MOR performance

Based on the above analysis and characterization of the structure and features of NiPc-NiPor COFs, it can be concluded that the MPc and MPor monomers in crystalline COFs form the well-defined, isolated, and atomically uniformly multiple single-metal active sites with different chemical environments, which is favorable for catalytic reaction. The electronic conductivity of NiPc-2HPor COF and NiPc-NiPor COF were performed by current (I)-voltage (V) measurements and electrochemical impedance spectrum (EIS) (Figs S14 and S15). We then calculated the conductivity values of all tested COFs from I-V test results by using a double probe system. As a result, the NiPc-NiPor COF exhibits higher specific conductivity values (7.28E-8 S m^−1^) than NiPc-2HPor COF (2.5E-8 S m^−1^). It can be concluded that the NiPc-NiPor COF possesses a superior electron transfer rate, which is due to their highly conjugated π-electron structure. Accordingly, NiPc-NiPor COF will be a more promising platform for electrocatalysis. Bearing the above ideas in mind, we then studied the ECR coupling MOR performances of NiPc-MPor COFs. The separated electrocatalysis tests were first conducted in a common H-cell reactor with a three-electrode standard system and the coupling reaction was then performed in the two-electrode system by electrochemical workstation. CO and HCOOH were detected as main the products for ECR and MOR with a minor by-product H_2_, which were quantified by gas chromatography (GC) and ion chromatography (IC) by external standard methods (Figs S16–S18), respectively.

**Figure 3.**
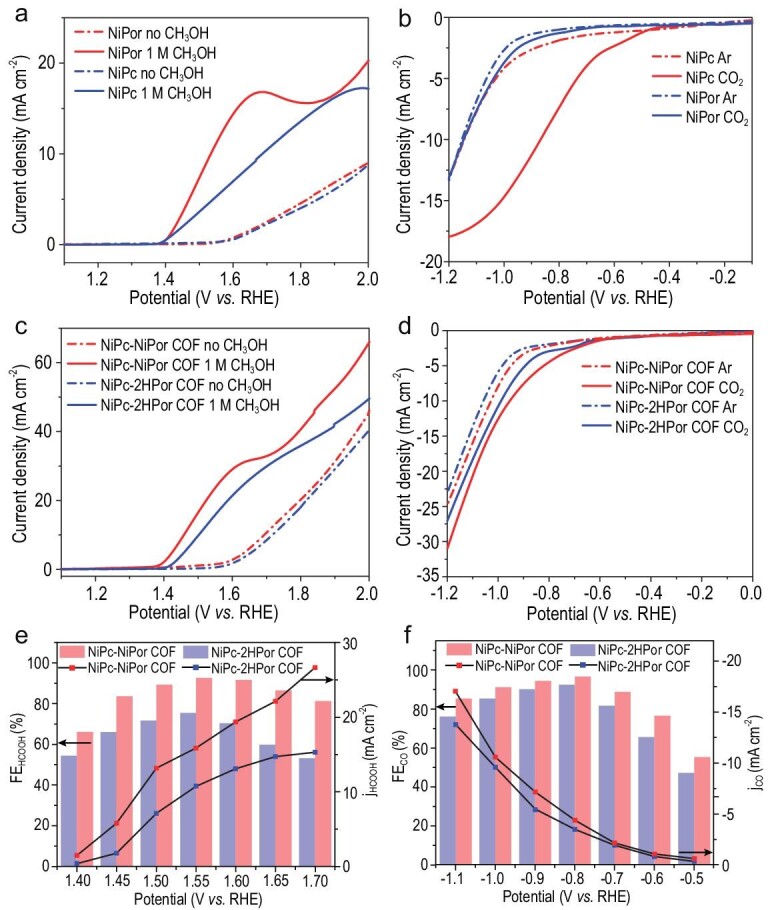
Electrocatalytic CO_2_ reduction and CH_3_OH oxidation performances of NiPc-MPor COFs. (a) LSV curves of NiPc and NiPor monomer for MOR. (b) LSV curves of NiPc and NiPor monomer for ECR. (c) LSV curves of NiPc-MPor COFs for MOR. (d) LSV curve of NiPc-MPor COFs for ECR. (e) FE and partial current density of NiPc-MPor COFs for MOR. (f) FE and partial current density of NiPc-MPor COFs for ECR.

First, we studied the NiPc and NiPor monomers as catalysts for MOR by conducting a linear sweep voltammetry (LSV) test on a three-electrode system in 1 M KOH electrolyte with or without methanol substrates, respectively. Interestingly, both NiPc and NiPor show effective enhanced current density for MOR in methanol electrolyte (Fig. 3a). Besides, NiPor monomers exhibit maximum current density when applied in a methanol electrolyte, suggesting that the NiPor may be a more effective active site for MOR. The LSV performance for ECR of NiPc and NiPor monomers also shows that NiPc has a higher current density than NiPor in CO_2_ (Fig. 3b), indicating that NiPc may play a key role in ECR. Based on the above results, it is reasonable to assume that integrating NiPc and NiPor monomers will be greatly beneficial to the ECR coupled with MOR performance.

Based on the above consideration, we then conducted the LSV test for NiPc-MPor COFs (Fig. 3). The results show that both NiPc-2HPor COF and NiPc-NiPor COF represent higher current density in methanol electrolyte compared with pure KOH electrolyte along with the increase in applied voltage (Fig. 3c and Fig. S19). Further, NiPc-NiPor COF shows more enhanced current intensity compared to NiPc-2HPor COF, which also indicates that the NiPor in the COF might play a key role in contributing to the activity of MOR. Besides, the Tafel slope of NiPc-NiPor COF at the anode in 1 M KOH with 1 M methanol is 123.84 mV dec^−1^, much lower than that in the pure KOH electrolyte (318.55 mV dec^−1^), suggesting that it has more favorable reaction kinetics for MOR (Fig. S20). We then tested the ECR performance for NiPc-MPor COFs as a cathode in Ar and CO_2_ saturated solution. Both the NiPc-NiPor COF and NiPc-2HPor COF obtain enhanced current density in the existence of CO_2_ compared to Ar environment, which suggests that the ECR is priority to HER process. Furthermore, the current density of NiPc-NiPor COF is almost same as that of NiPc-2HPor COF in a CO_2_ environment (Fig. 3d), which further illustrates that the NiPc (rather than NiPor) in COFs mainly contribute to ECR activity.

Encouraged by the above performance, we then explored, separately, the Faradaic efficiency (FE) and partial current density (j) of these samples for ECR or MOR on a three-electrode system in greater detail. We first discovered the NiPc and NiPor monomers under wide potentials ranging from −0.5 V to −1.1 V vs. RHE for cathode ECR and 1.4 V to 1.7 V vs. RHE for anode MOR and calculated the corresponding FE values (Figs S21 and S22). The results show that, for MOR, both the NiPc and NiPor monomers own effective FE_HCOOH_ and the detailed comparison suggests that the NiPor monomer has superior selectivity to NiPc. At the same time, based on the ECR performance as shown in Fig. S22, we can also conclude that the NiPc shows more superior selectivity for ECR. We then studied the NiPc-2HPor COF and NiPc-NiPor COF as catalysts for ECR and MOR tests, separately (Fig. 3e and f). Compared with NiPc-2HPor COF, NiPc-NiPor COF exhibits superior MOR catalytic activity and selectivity with maximal FE_HCOOH_ of up to 92.63% with a j_HCOOH_ of 15.84 mA cm^−2^ at 1.55 V vs. RHE. On the other hand, the NiPc-NiPor COF also shows the better activity than NiPc-2HPor COF on ECR, with maximal FE_CO_ of up to 96.57% and a partial current density (j_CO_) of −4.39 mA cm^−2^ at −0.8 V vs. RHE.

**Figure 4.**
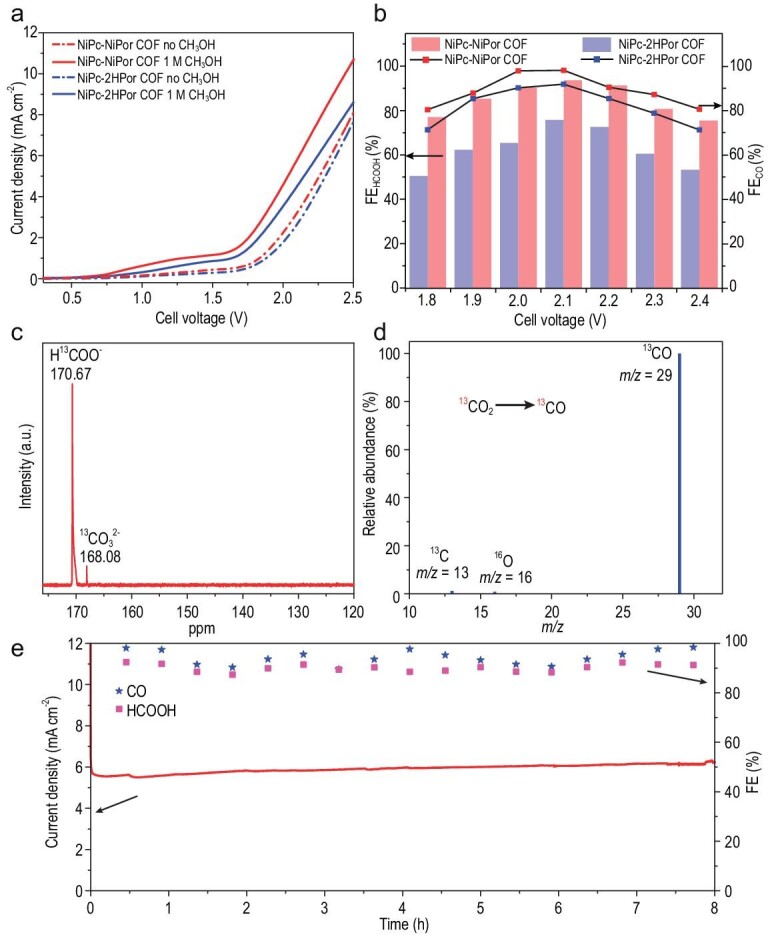
ECR and MOR coupling reaction performance for NiPc-MPor COFs. (a) LSV curves for the coupled ECR || MOR cell for NiPc-MPor COFs. (b) FE performances for the coupled ECR || MOR cell for NiPc-MPor COFs. (c) ^13^C-NMR spectra of the MOR electrolyte for NiPc-NiPor COF. (d) MS spectra of the ECR to CO for NiPc-NiPor COF. (e) Durability measurements of NiPc-NiPor COF at 2.1 V cell voltage.

Based on the above results, ECR and MOR coupling reaction performances were carried out by using a two-electrode H-cell, in which the NiPc-MPor COFs act as both cathode and anode active catalyst. Specifically, the anode part with 1 mg/cm^−2^ NiPc-MPor COFs active layer was applied in 1 M KOH electrolyte containing 1 M methanol, and the cathode with the same active material was applied in 0.5 M KHCO_3_ electrolyte (denoted as NiPc-MPor COFs || NiPc-MPor COFs). The LSV patterns for the paired MOR (1 M methanol) || ECR show that the NiPc-NiPor COF electrode only needs a cell voltage of 1.5 V to obtain a current density of 1.0 mA cm^−2^, which is much lower than the paired OER || ECR without methanol (Fig. 4a). We then tested the FE of two COFs under cell voltage ranging from 1.8 V to 2.4 V. When paired the MOR and ECR, the FE_CO_ of NiPc-NiPor COF exhibited higher than 90% in a potential range from 2.0 to 2.2 V and the maximum FE_CO_ can reach up to 98.12% with a partial current density (j_CO_) of ∼6.14 mA cm^−2^ at 2.1 V (Fig. 4b, and Figs S23 and S25). Meanwhile, NiPc-2HPor COF shows a little less FE_CO_ than the NiPc-NiPor COF for ECR, which can be concluded that the NiPc unit mainly contributed to ECR activity. On the other hand, NiPc-2HPor COF also shows much lower FE_HCOOH_ than NiPc-NiPor COF for MOR, which illustrates that the NiPor may play a key role for MOR. Besides, the corresponding anodic MOR produced HCOOH with FE over 75% at all applied voltages and toward a maximum value of 93.75% with a partial current density (j_HCOOH_) of ∼5.81 mA cm^−2^ at 2.1 V (Fig. 4b and Fig. S24). The detailed structure-functional relationships will be discussed will in the following part. To further detect the liquid product of NiPc-NiPor COF during MOR process in MOR || ECR cell, the reaction mixture was analyzed by ^1^H-NMR (Fig. S26). The CH_3_OH oxidation products, that is, HCOOH is obviously found in 8.27 ppm. For ECR, the ^1^H-NMR of reaction mixture shows no liquid products (Fig. S27), and no other except for CO and H_2_ is detected from GC and IC, which means CO and H_2_ are the only products of ECR. To verify the source of carbon atoms in HCOOH, we carried out isotope labeling. The MOR test was carried out in 1 M KOH electrolyte containing ^13^C labeled CH_3_OH, the ^13^C-NMR showed an evident peak of H^13^COOH as shown in Fig. 4c. The isotope labeling experiments were also performed to ascertain the carbon sources of ECR products, i.e. CO, ^13^CO (*m/z* = 29) was finally detected by gas chromatograph-mass spectrometer (GC-MS) (Fig. 4d). These results confirm that the produced HCOOH and CO originated from the reactant CH_3_OH and CO_2_, respectively, instead of decomposition of the catalyst. The durability of a catalyst is also one of the most important factors in further practice application. Therefore, we then evaluated the stability of NiPc-NiPor COF in the electrochemical conditions by chronoamperometric testing (Fig. 4e). After long-time evaluation, no obvious decay in FE and current density was detected during 8.5 h (the FE of CO and HCOOH was analyzed every 0.5 h). Furthermore, the crystalline structure was preserved from the PXRD patterns of NiPc-NiPor COF after being immersed in 1 M KOH aqueous solution and 0.5 M KHCO_3_ aqueous solution for 48 h, respectively (Fig. S28). More importantly, the PXRD patterns show that NiPc-NiPor COF still maintains crystallinity after the electrocatalytic reaction (Fig. S29). It is noticed that the PXRD peak intensity after electrocatalytic tests on anode showed reduced. This phenomenon maybe caused by the intrinsic instability of this COF under the combination of strong base and electric field conditions, the electrochemical corrosion by electrolysis, and mechanical force by stirring. All the above results confirm that these COFs are highly stable catalysts.

### Investigating structure-functional relationships

**Figure 5.**
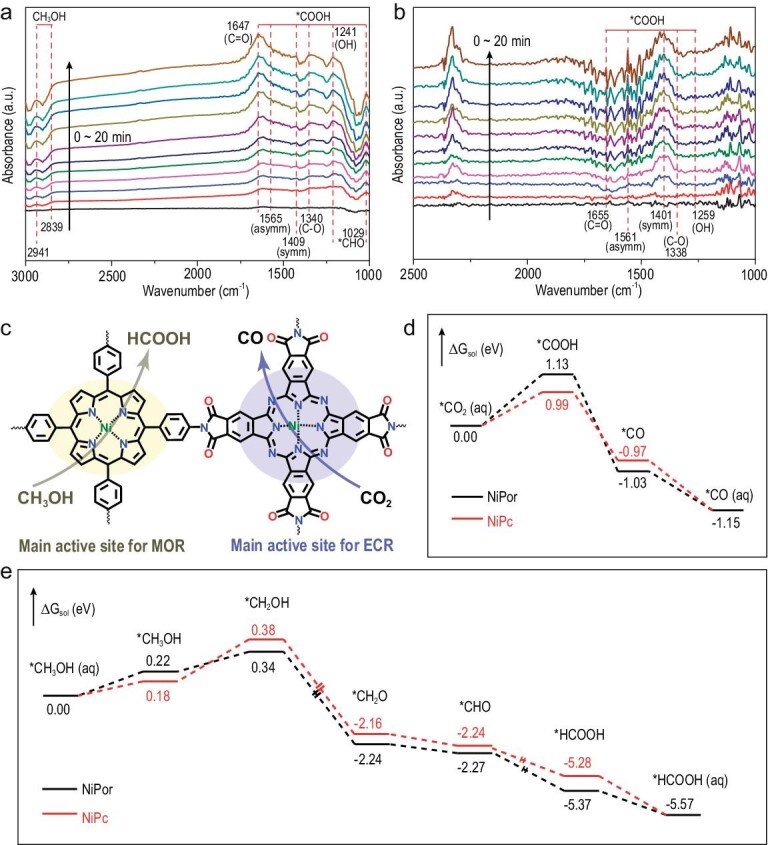
*In-situ* FT-IR and DFT calculations of the catalytic process. (a) *In-situ* FT-IR spectra for MOR using NiPc-NiPor COF as catalyst. (b) *In-situ* FT-IR spectra for ECR using NiPc-NiPor COF as catalyst. (c) Schematic of the mechanism of NiPc-NiPor COF for ECR coupled with MOR. (d) Free energy diagram (FED) of NiPc and NiPor for ECR. (e) FED of NiPc and NiPor for MOR.

We then performed the *in-situ* FT-IR investigation of the catalytic process to study the key intermediates for ECR and MOR. For the MOR process (Fig. 5a), an increasing positive band centers at ∼1647 cm^−1^ which corresponds to the C=O of *COOH and is clearly observed in applied cell potential of 1.55 V vs. the RHE. Meanwhile, a small positive band centers at 1565, 1409, 1340 and 1241 cm^−1^, which corresponds to the asymmetry and symmetry stretch of C-O and OH vibrations for *COOH also observed [45]. Besides, the increasing peak at 1029 cm^−1^ suggests that *CHO species exist [46]. The above results show that the *COOH and *CHO are the key intermediates for CH_3_OH oxidation to HCOOH. In addition, the bands at 2941 and 2839 cm^−1^ in the spectra are ascribed to surface CH_3_OH species. As for the ECR process (Fig. 5b), the *COOH is also observed as a key intermediate for CO_2_ reduction to CO, whose peaks appear at 1700–1200 cm^−1^ [47].

Guided by the *in-situ* FT-IR analysis and conclusions, we further investigated the ECR and MOR catalytic processes in detail based on DFT theoretical calculations (Fig. 5c). For the ECR process, the electron transfer to the adsorbed CO_2_ was then combined with a H proton to generate *COOH which is calculated to be the rate determining step (RDS) on NiPc-NiPor COF. Interestingly, the Gibbs free energy on NiPc for the RDS step is 0.99 eV, which is obviously smaller than the process on NiPor (Fig. 5d). Therefore, based on the minimum energy principle, the ECR process is more likely to occur on the NiPc part. As for the MOR process, the RDS is determined to be the oxidation process of *CH_3_OH to *CH_2_OH. It is noted that the energy barrier for *CH_3_OH to *CH_2_OH on NiPor and NiPc have a small difference, this indicated that the MOR catalytic process can occur in both NiPor and NiPc. From the calculation results, we also found that the free energy for *CH_3_OH to *CH_2_OH process on NiPor is 0.34 eV, which is slightly weaker compared to NiPc (0.38 eV) (Fig. 5e). Thus, we can conclude that the main active site for MOR contributed to the NiPor part and simultaneously conjugated with NiPc, these synergistic effects caused significant catalytic activity during the MOR reaction.

## CONCLUSION

In conclusion, we rationally designed and synthesized two stable PI phthalocyanine-porphyrin bifunctional COFs in pure water by a hydrothermal method for electrocatalytic cathodic CO_2_ reduction coupled with anodic CH_3_OH oxidation. The dual Ni sites in NiPc-NiPor COF in different chemical environments are mainly devoted to different electrocatalytic reactions (i.e. MOR and ECR). Interestingly, NiPc-NiPor COF behaves as the superior FE and j for both MOR and ECR. In detail, the NiPc-NiPor COF shows FE_CO_ = 98.12% (j_CO_ = 6.14 mA cm^−2^) for ECR and FE_HCOOH_ = 93.75% (j_HCOOH_ = 5.81 mA cm^−2^) for MOR. According to exhaustive electrochemical measurement and comparison results, we demonstrate that the NiPc unit mainly contributes to the ECR with the assistance of NiPor, meanwhile NiPor is mainly for the MOR process and conjugates with NiPc. These ingenious synergistic effects cause significant catalytic activity for ECR coupled MOR reaction. More importantly, an in-depth mechanistic study based on *in-situ* FT-IR and DFT simulation also confirmed the above conclusions. Our work provides a new insight into the design and development of dual functional COFs-based catalysts for various catalytic reactions.

## METHODS

### Synthesis of PI-linked metallophthalocyanine-metalloporphyrin COFs

Synthesis of NiPc-2HPor COF: A Pyrex tube measuring 10 × 200 mm (o.d × length) was charged with NiPc (23.1 mg), 2HPor (16.8 mg), H_2_O (2.5 mL). After sonication for about 60 minutes, the tube was then flash frozen at 77 K (liquid N_2_ bath) and degassed by three freeze-pump-thaw cycles, and refilled by N_2_ (99.999%) to 1 bar then flame sealed. Then, warmed to room temperature, the mixture was heated at 230°C and left undisturbed for 48 h. A black precipitate was isolated by filtration in a Buchner funnel and was washed with THF and acetone until the filtrate was colorless. The wet sample was transferred to a Soxhlet extractor and washed with THF for 24 hours. Finally, the product was evacuated at 120°C under dynamic vacuum overnight to yield the activated sample (∼21 mg, 57% yield).

Synthesis of NiPc-NiPor COF: The NiPc-NiPor COF was synthesized by post synthesis method. In detail, NiPc-2HPor COF (15 mg) and Ni(OAc)_2_·2H_2_O (50 mg) were added to the ethanol (20 mL). After being purified by N_2_, the mixture was heated and refluxed for 12 h at N_2_ atmosphere. Following that, the solutions were cooled down to room temperature and filtered. The filter cake was washed thoroughly with water and ethanol to remove free metal ions. The final filter cake was dried at 120°C under dynamic vacuum overnight to get NiPc-NiPor COF (∼14 mg, 82% yield).

### Electrochemical measurements

Electrocatalytic ECR coupling MOR experiments: All electrochemical tests were applied in an airtight H-cell (Tianjin Aida Hengsheng Technology, China) which separated the cathodic and anodic chambers by using Nafion 117 membrane. The standard two-electrode system, i.e. catalyst-modified carbon fiber papers both as working anode and cathode electrode, and the ECR coupling MOR tests on the electrochemical workstation (Bio-Logic VSP) and the CO_2_ saturated 0.5 M KHCO_3_ and 1 M CH_3_OH in 1 M KOH were used as electrolyte. The potential range of 1.8 to 2.4 V (cell voltage, step size = 0.1 V) was applied during the ECR coupling MOR test and calculated the Faradaic efficiency and current density. The yield of CO and H_2_ was quantified by gas chromatography (GC-7920, CEAulight, China). The HCOOH yield was quantified by ion chromatography (Ion Chromatography System, Themorpher, China). The working electrode was similar to the preparation of ECR. The polarization curve results were obtained by performing linear sweep voltammetry (LSV) mode with a scan rate of 5 mV s^−1^. Potentials were measured against an Ag/AgCl reference electrode and the results were converted to those against a reversible hydrogen electrode (RHE) based on the RHE calibration. Electrochemical impedance spectroscopy (EIS) measurement was performed on the electrochemical analyzer in a frequency range from 100 kHz to 100 mHz by applying an AC voltage with 10 mV.

## Supplementary Material

nwad226_Supplemental_File
