# Supplementary material for: Green synthesis of bifunctional phthalocyanine-porphyrin COFs in water for efficient electrocatalytic CO2 reduction coupled with methanol oxidation
Source: Natl Sci Rev. 2023 Sep 2;10(11):nwad226. doi: 10.1093/nsr/nwad226 (PMC10561706; doi:10.1093/nsr/nwad226)
Supplement: nwad226_Supplemental_File [file nwad226_supplemental_file.pdf]

Supplementary Information

Green Synthesis of Bifunctional Phthalocyanine-porphyrin COFs in Water for Efficient Electrocatalytic CO_2 Reduction Coupled with Methanol Oxidation

Mi Zhang,[†] Jia-Peng Liao,[†] Run-Han Li,[†] Sheng-Nan Sun, Meng Lu, Long-Zhang Dong, Pei Huang, Shun-Li Li, Yue-Peng Cai, and Ya-Qian Lan*

School of Chemistry, South China Normal University, Guangzhou, 510006, China.

[*]*Corresponding author. E-mail: yqlan@m.scnu.edu.cn*

[†]*Equally contributed to this work.*

Table of Contents

S1. Material and Characterization methods 3

S1.1. Materials and Synthetic procedures..........3

S1.2. Powder X-ray diffraction..........5

S1.3. Thermogravimetric analysis..........6

S1.4. X-ray photoelectron spectroscopy..........8

S1.5. Transmission electron microscopy..........11

S1.6. Scanning electron microscopy..........12

S1.7. Electro-conductivity measurements..........13

S2. Electrocatalytic Experiments..........14

S2.1. Electrocatalytic CO_2 reduction (ECR) experiments..........14

S2.2. Electrocatalytic CH_3OH oxidation (MOR) experiments..........15

S2.3. Electrocatalytic ECR coupling MOR experiments..........19

S2.4. Analysis of liquid products..........21

S2.5. Stability test..........22

S3. Computational methods..........23

S3.1. DFT calculations..........23

S3.2. Structural modeling..........24

S1.1 Materials and Synthetic procedures

Materials

All solvents and reagents obtained from commercial sources were used without further purification. 2,3,9,10,16,17,23,24-octacarboxylphthalocyaninato Nickel (NiPc) and 5,10,15,20-tetrakis(para-aminophenyl) porphyrin (2HPor) were purchased from Shanghai Kylpharm Co., Ltd. N, N-Dimethylformamide (DMF, AR), chloroform, tetrahydrofuran (THF, 99.5%), methanol, ethanol and $Ni(OAc)_2 \cdot 4H_2O$ were purchased from Sinopharm Chemical Reagent Co., Ltd.

Characterization.

The powder X-ray diffraction (PXRD) spectra were recorded on a Bruker D8 Advance diffractometer with Cu Kα radiation (λ = 1.5418 Å) at 40 kV, 40 mA. Diffraction intensity data for 2θ from 3 ~ 40° were collected at the 2θ step increment of 0.01 °. Fourier-transform infrared (FT-IR) spectra of starting materials and COF samples were recorded in a Thermo Nicolet IS50 FT-IR spectrometer under ambition conditions. The surface morphology of catalysts was collected using high resolution thermal field emission scanning electron microscope (SEM, JSM-7600 F) with an acceleration voltage of 10 kV. Transmission electron microscopy (TEM), high-resolution TEM (HRTEM) and energy dispersive X-ray spectroscopy (EDX) were obtained on a JEOL 2100F microscopy at an accelerating voltage of 200 kV. X-ray photoelectron spectroscopy (XPS) were recorded using an ESCALAB Xi^+ from Thermo Scientific equipped with an Al Kα micro focused X-ray source and the C1s peak at 284.6 eV as internal standard. Nitrogen sorption and apparent surface areas was measured at 77.3 K and CO_2 sorption were measured at 273 K using an Autosorb IQ2 absorptiometer (Quantachrome Instruments) volumetric adsorption analyzer. COFs samples were degassed at 150 ℃ for 12 h on analysis port under vacuum before test. Pore size distributions of COFs were calculated using nonlocal density functional theory (NL-DFT) model. Thermogravimetric analysis of COFs powder samples was performed on a Diamond TG/DTA/DSC Thermal Analyzer System (Perkin-Elmer, USA) with heating rate of 10 ℃ min^{-1} to 900 ℃ under N_2 atmosphere and 10 ℃ min^{-1} to 900 ℃ under air, respectively. The isotopelabeled experiment was performed using $^{13}CO_2$ and $^{13}CH_3OH$ instead of $^{12}CO_2$ and $^{12}CH_3OH$, the result was analyzed by GC-MS (7890A and 5875C, Agilent) and ^{13}C-NMR, respectively. ^{1}H-NMR and ^{13}C-NMR were carried out on AVANCE III 400M spectrometer (Bruker). Ag/AgCl electrode and Pt wire (or carbon rod) were used as reference electrode and counter electrode, respectively. CO_2-saturated 0.5 M $KHCO_3$ solution and Ar-saturated 1 M KOH solution containing 1 M methanol were used as electrolyte for ECR and MOR, respectively. In-situ FT-IR spectra were recorded during stepping the working electrode potential. The relatively direct current conductivity tests were conducted with a probe station at room temperature (25 ℃) under ambient conditions with a computer-controlled analog-to-digital converter (keysight B2902B). The in-situ FT-IR study was carried out on a NICOLET iS50 FT-IR spectrometer equipped with an MCT detector cooled with liquid nitrogen. The Au-coated Si crystal (20 mm in diameter, MTI Corporation) was used as the conductive substrate for catalysts and the IR reflection element. The catalysts suspensions were dropped on the Au/Si surface as the working electrode. Ag/AgCl electrode and Pt wire (or carbon rod) were used as reference electrode and counter electrode, respectively. CO_2-saturated 0.5 M $KHCO_3$ solution and Ar-saturated 1 M KOH

solution containing 1 M methanol were used as electrolyte for ECR and MOR, respectively. In-situ FT-IR spectra were recorded during the stepping of the working electrode potential.

Synthesis of 5,10,15,20-tetrakis(para-aminophenyl) porphyrin Nickel (II) (NiPor).

NiPor was synthesized according to a previously published procedure with some modified[1]. In a 250 ml three-neck round bottomed falsk, 2HPor (200 mg, 0.3 mmol), $Ni(OAc)_2 \cdot 4H_2O$ (299 mg, 1.2 mmol) was added, after purified by high purity nitrogen for three times by using a Schlenk equipment, a premixed solvent of methanol (20 mL), chloroform (90 mL) and DMF (30 mL) were added carefully. The solution was heated to 80 ℃ under stirring under nitrogen for 24 h. After cooled to room temperature, the solution was transferred into a separatory funnel and washed with water (3 × 100 mL). The organic layer was collected and dried over Na_2SO_4. The solvent was removed by rotary evaporation to give NiPor as a dark purple solid (~150 mg, 69% yield).

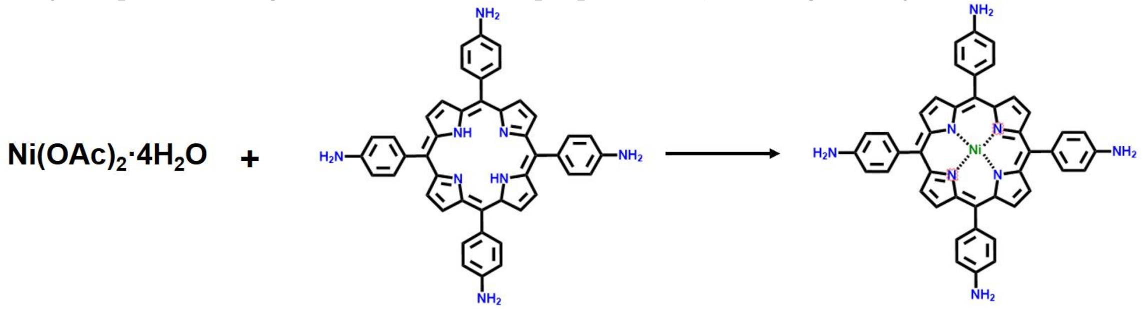

Scheme S1. Schematic representation of the synthesis procedure and structure of NiPor.

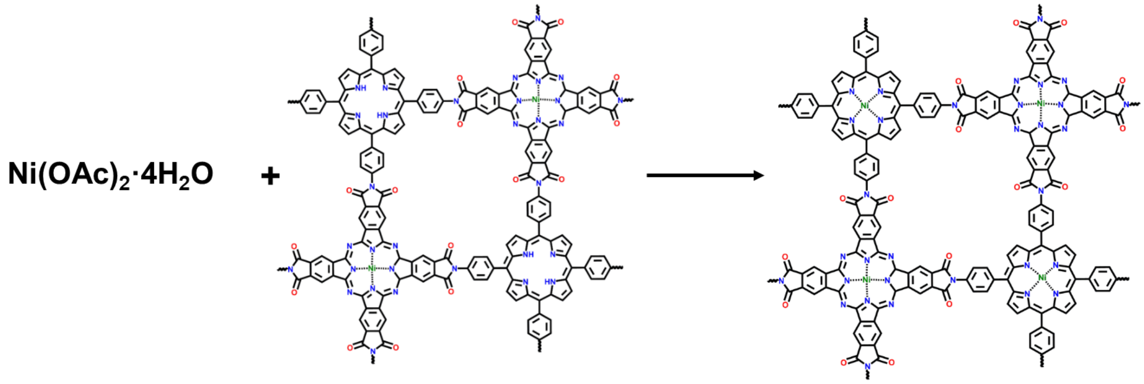

Scheme S2. Schematic representation of the synthesis procedure and structure of NiPc-NiPor COF.

S1.2. Powder X-ray diffraction

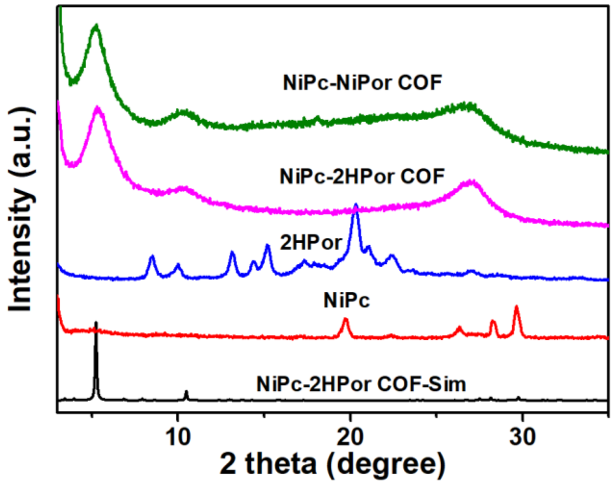

Figure S1. Comparison of PXRD patterns of NiPc-2HPor COF and NiPc-NiPor COF with 2HPor and NiPc.

S1.3. Thermogravimetric analysis

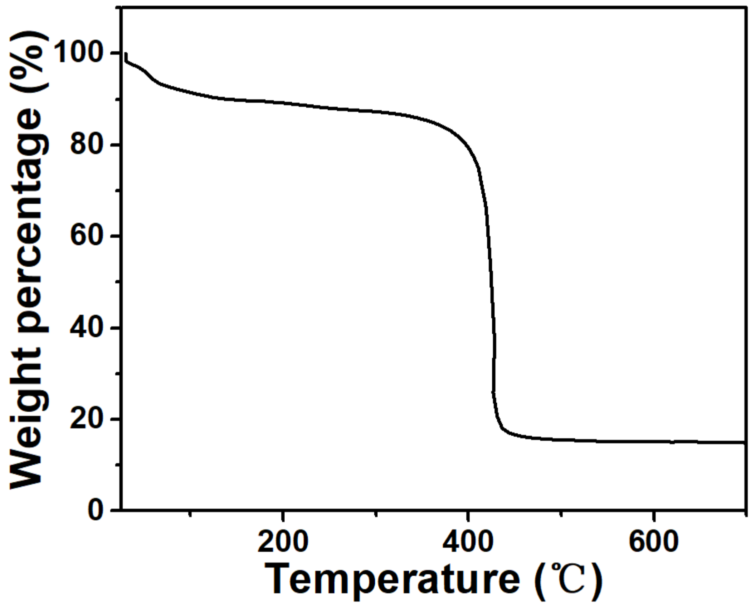

Figure S2. TGA for the NiPc-2HPor COF under O_2 atmosphere.

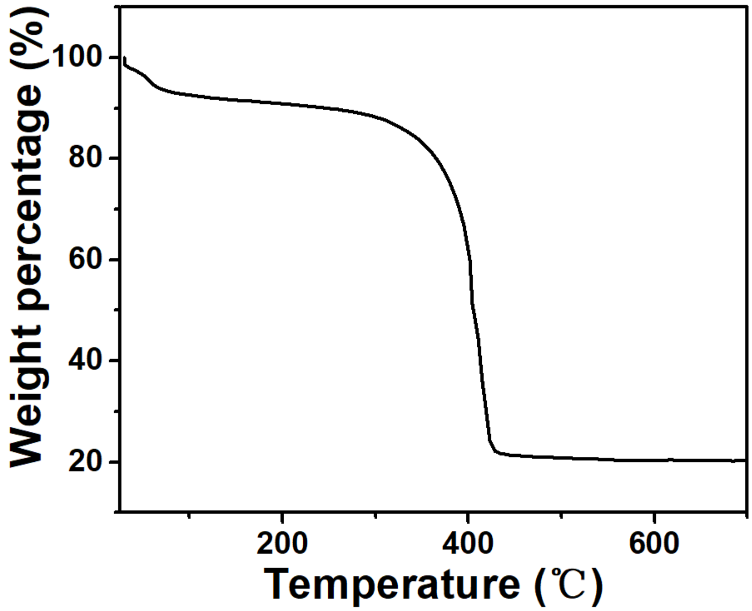

Figure S3. TGA for the NiPc-NiPor COF under O_2 atmosphere.

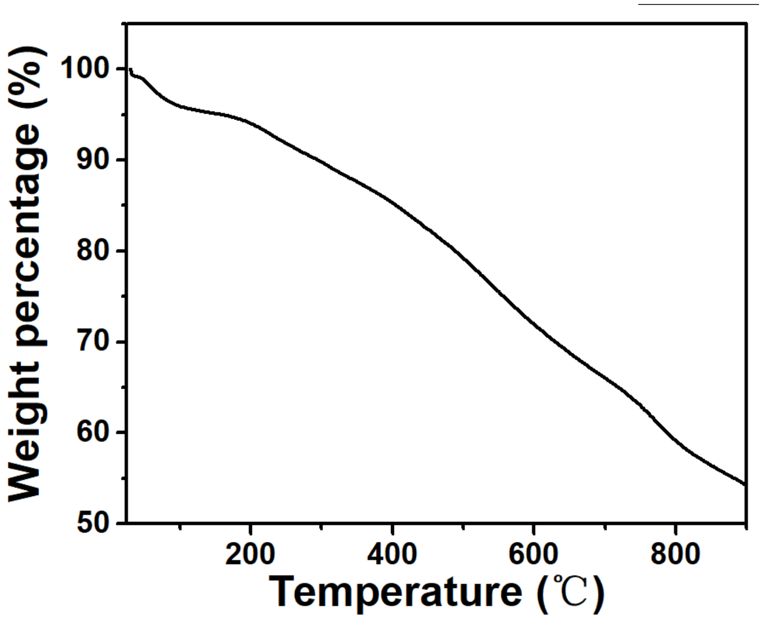

Figure S4. TGA for the NiPc-2HPor COF under N_2 atmosphere.

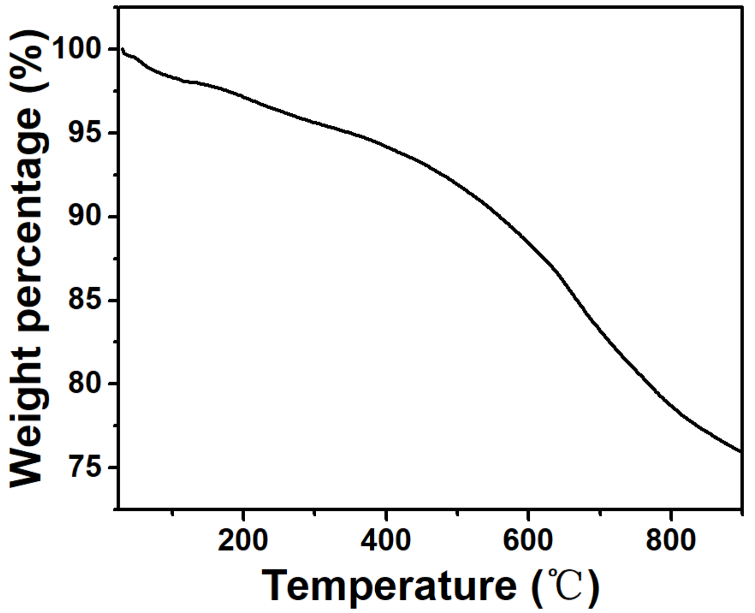

Figure S5. TGA for the NiPc-NiPor COF under N_2 atmosphere.

S1.4. X-ray photoelectron spectroscopy

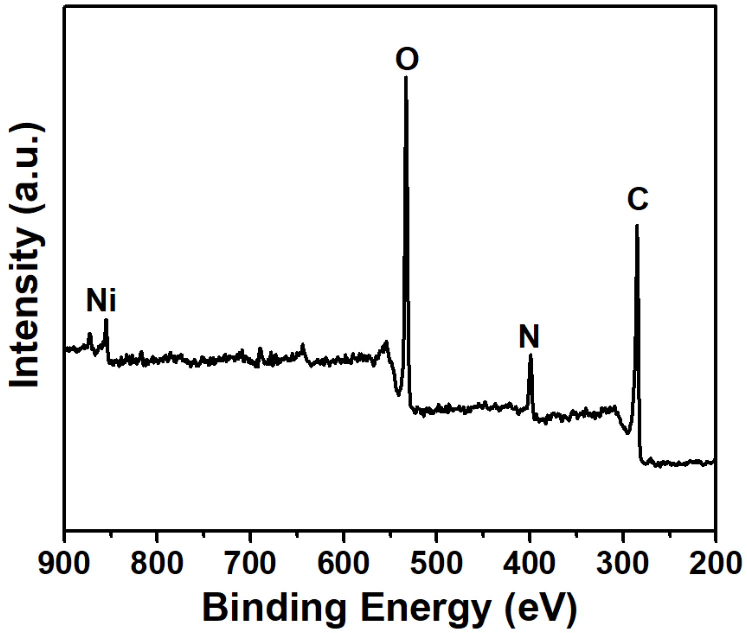

Figure S6. Survey scan XPS profiles of NiPc-2HPor COF.

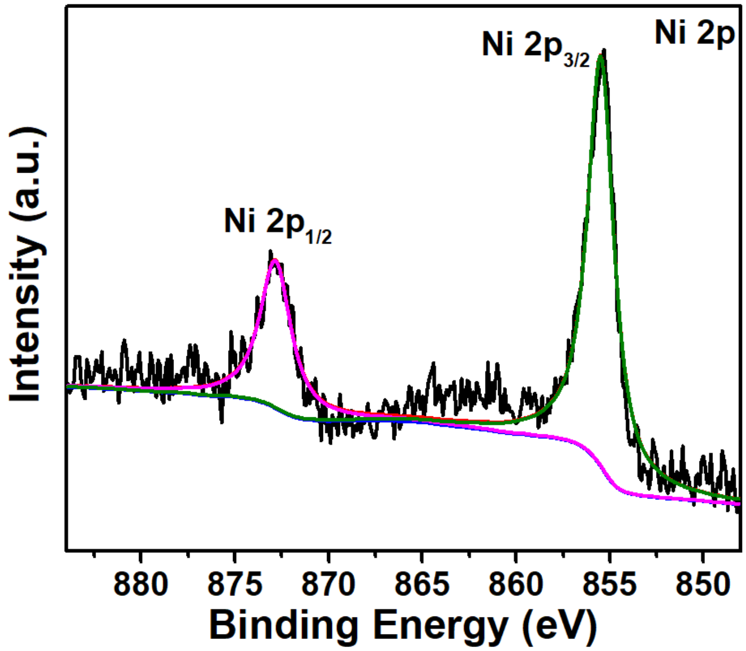

Figure S7. High resolution Ni 2p XPS profiles of NiPc-2HPor COF.

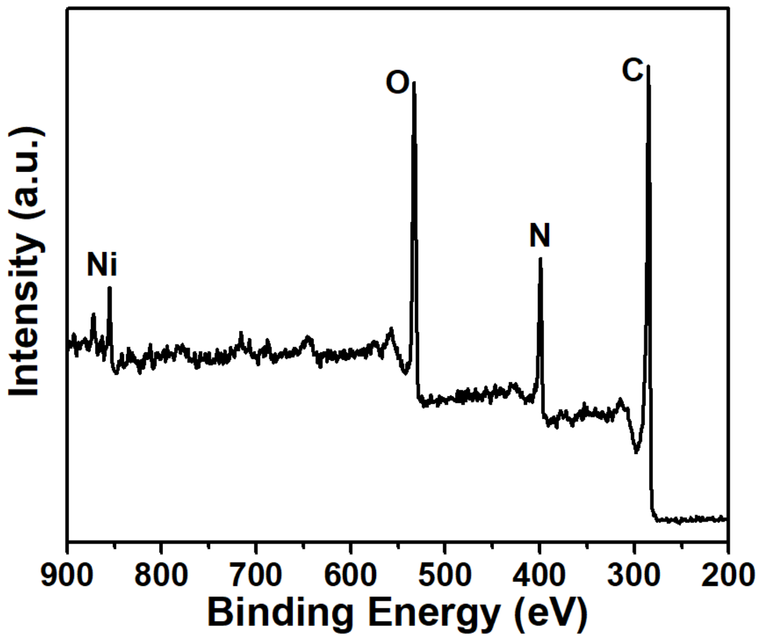

Figure S8. Survey scan XPS profiles of NiPc-NiPor COF.

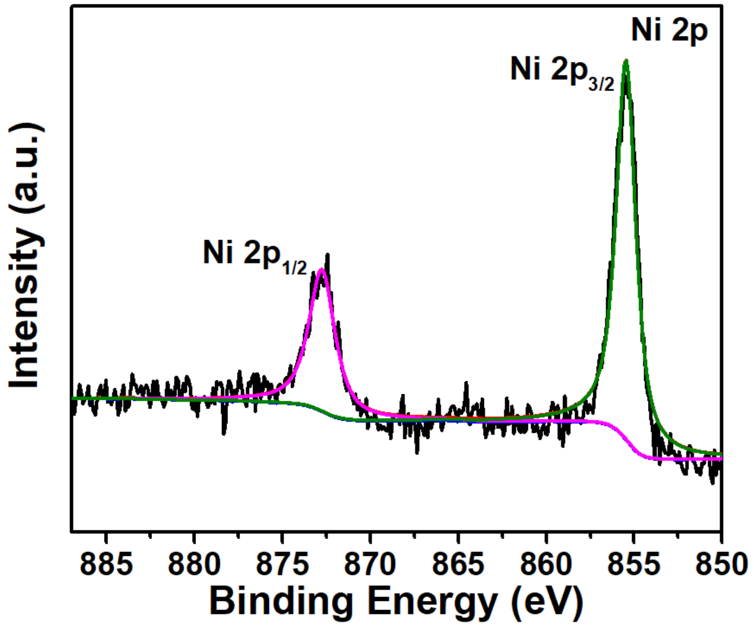

Figure S9. High resolution Ni 2p XPS profiles of NiPc-NiPor COF.

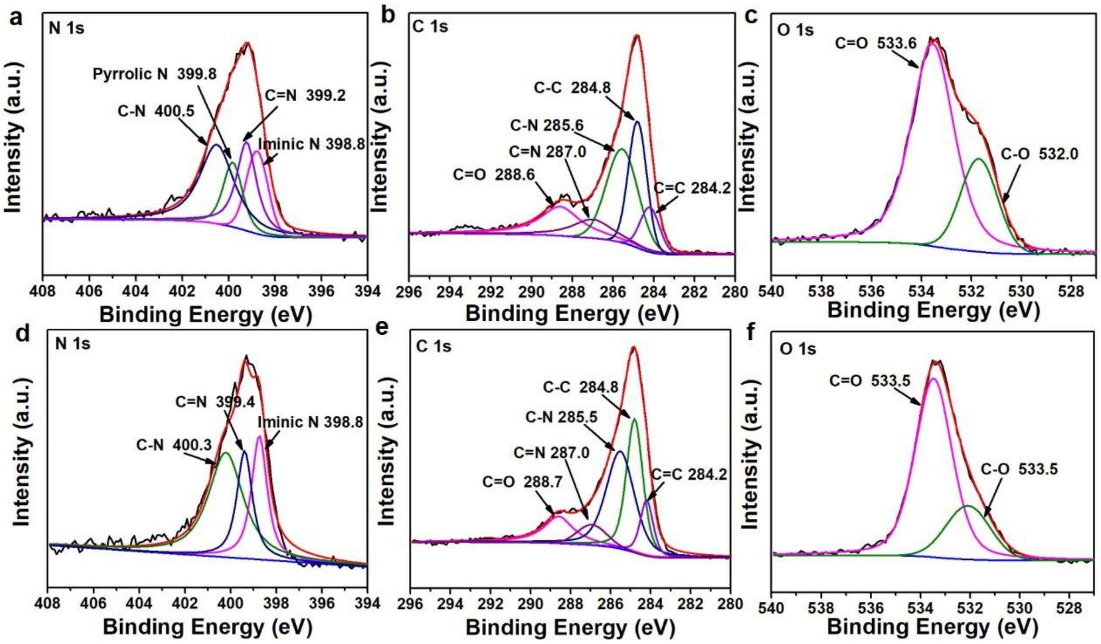

Figure S10. High resolution N 1s, C 1s and O 1s XPS spectrum and deconvolution results for NiPc-2HPor COF (a, b and c) and NiPc-NiPor COF (d, e, and f).

Table S1. XPS content tables of N 1s for NiPc-2HPor COF and NiPc-NiPor COF.

Sample	N species contribution (%)			
	C-N	Pyrrolic N	C=N	Iminic N
NiPc-2HPor COF	39.3	26.0	18.9	15.8
NiPc-NiPor COF	35.6	0	23.5	40.9

S1.5. Transmission electron microscopy

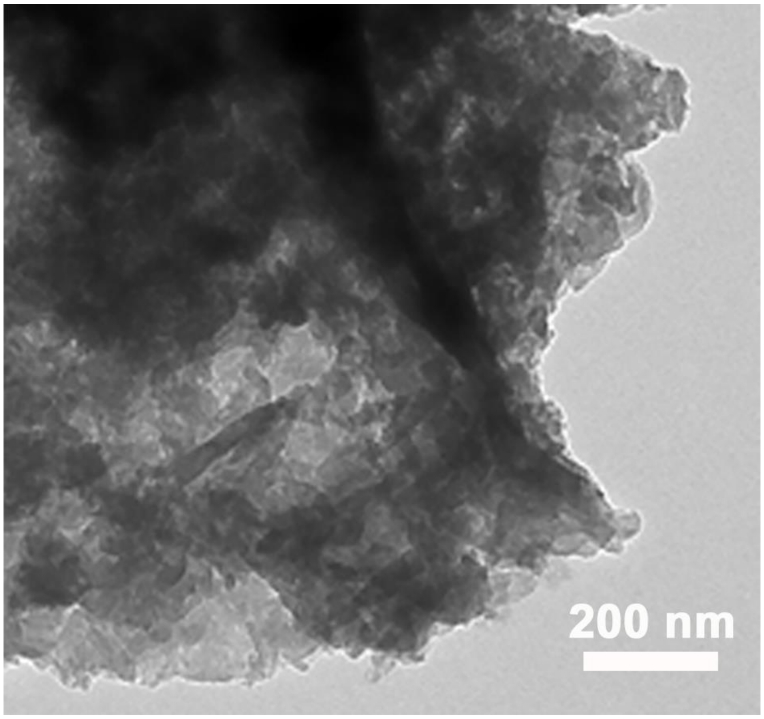

Figure S11. TEM image of NiPc-2HPor COF.

S1.6. Scanning electron microscopy

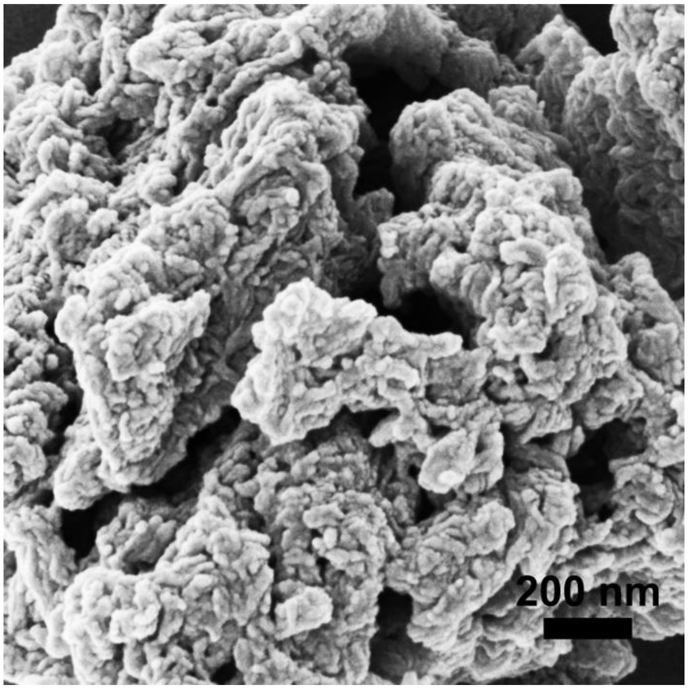

Figure S12. SEM image of NiPc-2HPor COF.

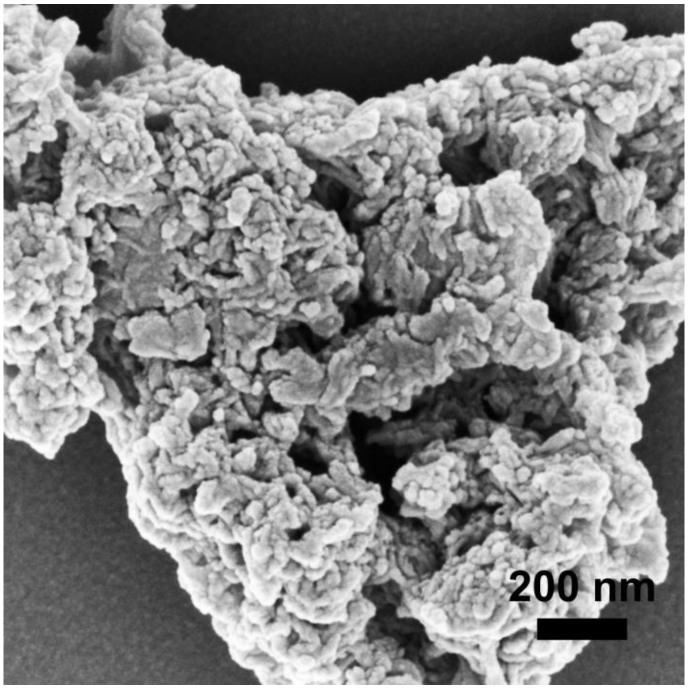

Figure S13. SEM image of NiPc-NiPor COF.

S1.7. Electro-conductivity measurements

The conductivity values of all tested COFs were calculated based on Eq. (1) as below.

$$\sigma = L/(R*S) = L/(R*\pi*r^2) \qquad \text{Eq. (1)}$$

where σ: conductivity, S m^{-1}
L: thickness of pellet, m
R: resistance value, Ω
S: cross section area, m^2
r: radius, m

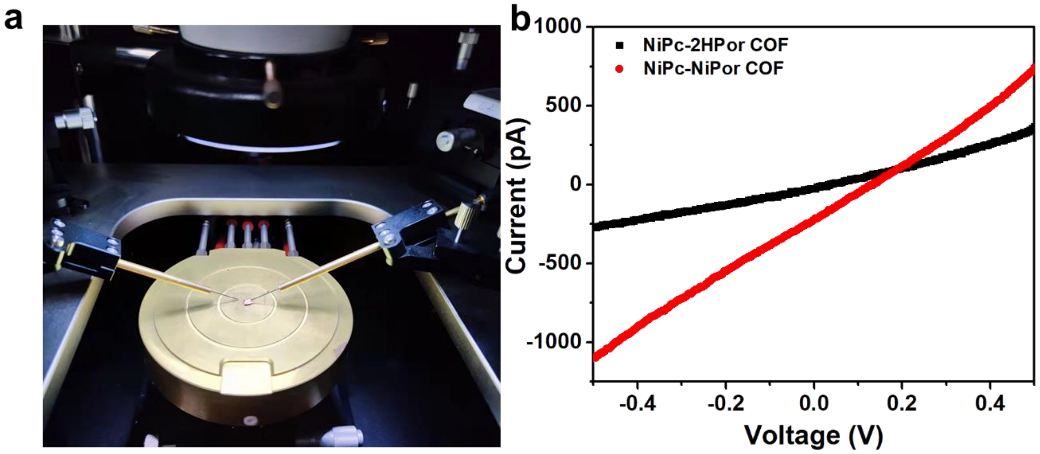

Figure S14. Electronic conductivity test of NiPc-2HPor COF and NiPc-NiPor COF. (a) The photograph of I-V measurement device. (b) I-V curve of NiPc-2HPor COF and NiPc-NiPor COF.

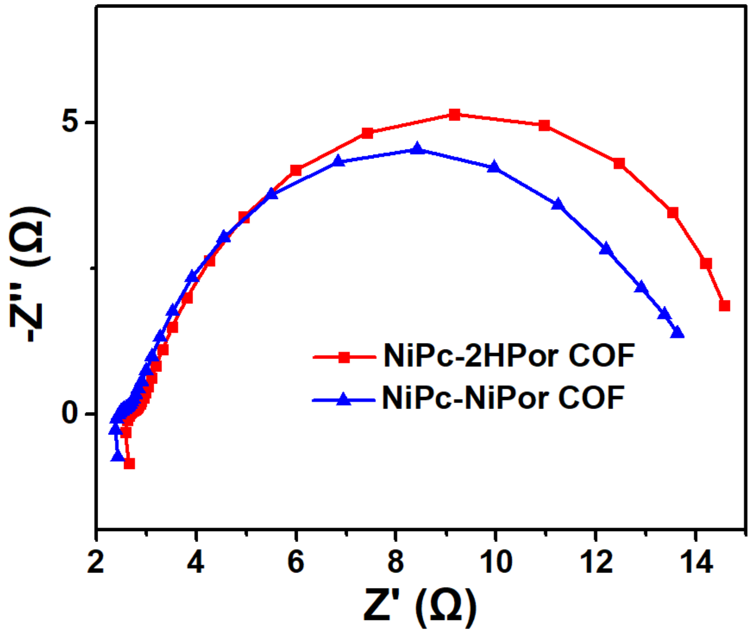

Figure S15. EIS spectra of NiPc-2HPor COF and NiPc-NiPor COF in 1 M KOH

solution.

S2. Electrocatalytic Experiments

S2.1. Electrocatalytic CO_2 reduction (ECR) experiments

The ECR performance evaluation was conducted based on our previous works [2]. All electrochemical tests were applied in an air tight H-cell (Tianjin Aida Heng sheng Technology, China) with separated the cathodic and anodic chambers by Nafion 117 membrane. The standard three-electrode system, i.e. catalyst-modified carbon fiber papers as working electrode with Ag/AgCl as reference electrode and carbon as counter electrode was used to conduct the ECR tests on the electrochemical workstation (Bio-Logic VSP) and the CO_2 saturated 0.5 M $KHCO_3$ was used as electrolyte. The potential range of -0.5 to -1.1 V vs. RHE (step size = 0.1 V) was applied during the ECR test and calculated the FE_{CO} and current density. The yield of CO and H_2 was quantified by gas chromatography (GC-7920, CEAulight, China).

The polarization curves results were obtained by performing linear sweep voltammetry (LSV) mode with a scan rate of 5 mV s^{-1}. Potentials were measured against an Ag/AgCl reference electrode and the results were converted to those against a reversible hydrogen electrode (RHE) based on the RHE calibration.

The working electrode was prepared as follows: 10 mg of ground samples and 10 mg of acetylene black (AB) were mixed by grinding. Then the mixture was added to a 1 mL solution containing ethanol (500 μL) water (400 μL) and Nafion solution (5 wt%, 100 μL) to produce a black suspension. After sonicating for 30 min, 50 μL uniformly mixed suspension was directly spray-coated on a hydrophobic carbon paper (1 × 2 cm) to form a 1 × 1 cm^2 catalyst area with a catalyst loading density of ~1 mg cm^{-2}.

The FE_{CO} was calculated according to the following equation:

$$FE_{products} = \frac{N \times F \times n_{products}}{Q} \times 100\%$$

Where N is the number of electrons transferred for products. (N = 2 for CO_2 to CO conversion and H_2O to H_2 conversion), F is the Faraday constant (96485 C mol^{-1}), $n_{products}$ is the moles of produced products (mol), Q is the total charge obtained from chronoamperometry (C).

S2.2. Electrocatalytic CH_3OH oxidation (MOR) experiments

The MOR performance evaluation was conducted similarly to the ECR, except the 1 M CH_3OH in 1 M KOH was used as electrolyte. The potential range of 1.4 V to 1.7 V vs. RHE (step size = 0.05 V) was applied during the ECR test and calculated the FE_{HCOOH} and current density. The yield HCOOH was quantified by ion chromatography (Ion Chromatography System, Thermo Fisher Scientific). The working electrode was similar to the preparation of ECR.

The polarization curves results were obtained by performing linear sweep voltammetry (LSV) mode with a scan rate of 5 mV s^{-1}. Potentials were measured against an Ag/AgCl reference electrode and the results were converted to those against a reversible hydrogen electrode (RHE) based on the RHE calibration.

The FE_{HCOOH} was calculated according to the following equation：

$$FE_{products} = \frac{N \times F \times n_{products}}{Q} \times 100\%$$

Where N is the number of electrons transferred for products. (N = 4 for CH_3OH to HCOOH conversion), F is the Faraday constant (96485 C mol^{-1}), $n_{products}$ is the moles of produced products (mol), Q is the total charge obtained from chronoamperometry (C).

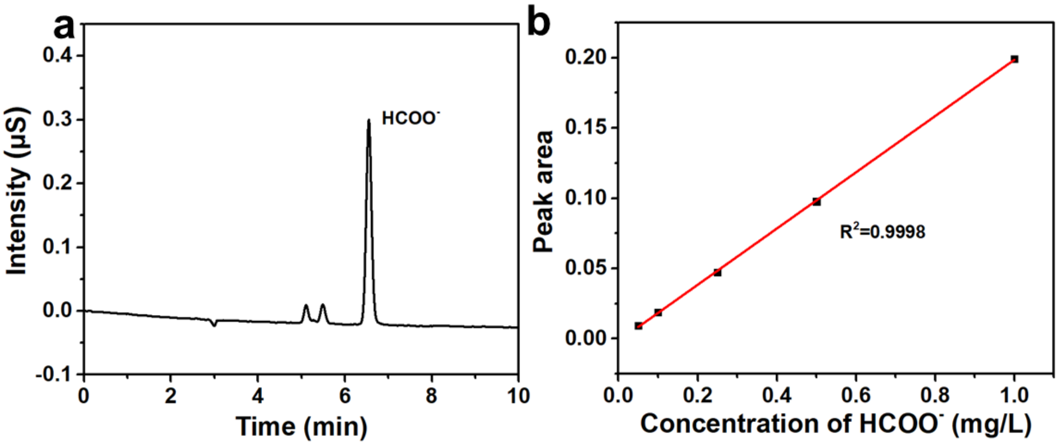

Figure S16. (a) The Ion chromatographic spectrum of MOR product (HCOOH). (b) Standard curve of MOR product (HCOOH).

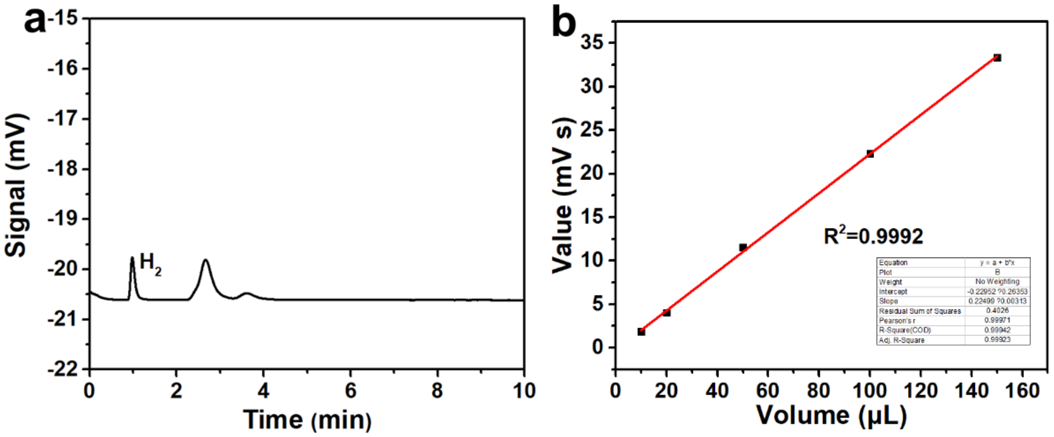

Figure S17. (a) The gas chromatography of ECR product (H_2). (b) Standard curve of ECR product (H_2). The gas used to make the standard curve is commercially available.

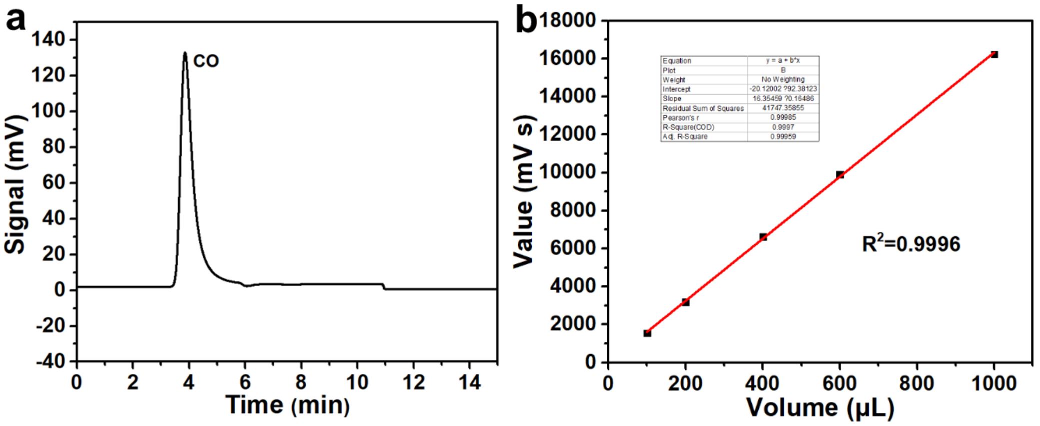

Figure S18. (a) The gas chromatography of ECR product (CO). (b) Standard curve of ECR product (CO). The gas used to make the standard curve is commercially available.

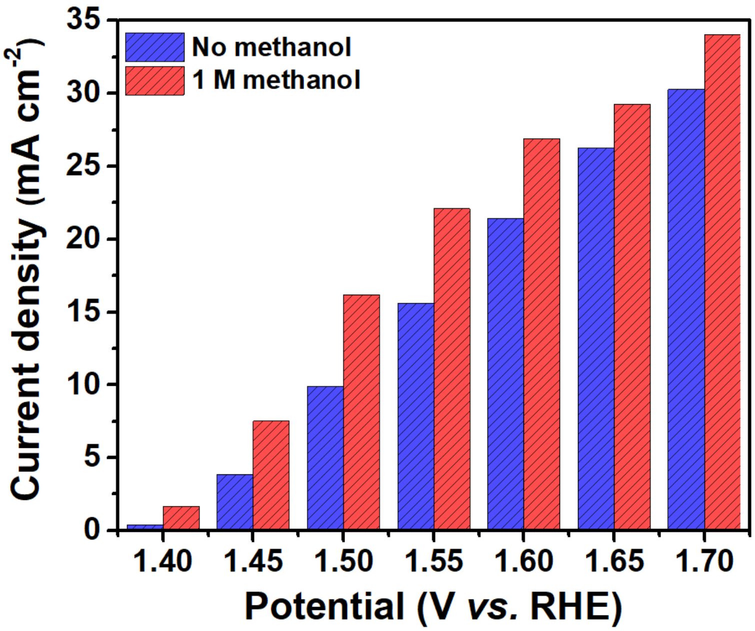

Figure S19. Current densities at varied anodic potentials (1.4 V, 1.45 V, 1.5 V, 1.55 V, 1.6 V, 1.65 V and 1.7 V vs. RHE) by NiPc-NiPor COF in 1 M KOH with and without the addition of 1 M methanol.

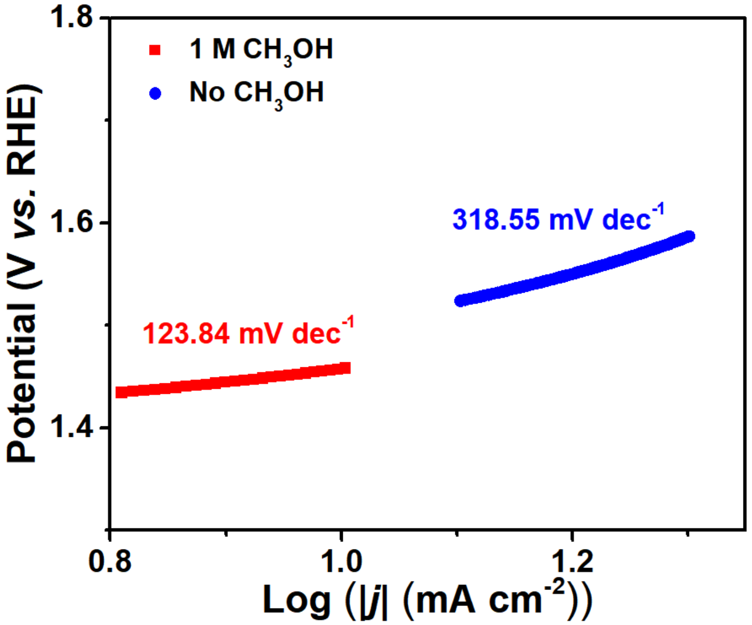

Figure S20. Tafel plots of NiPc-NiPor COF at anode in 1 M KOH with and without the addition of 1 M methanol derived from the LSV results.

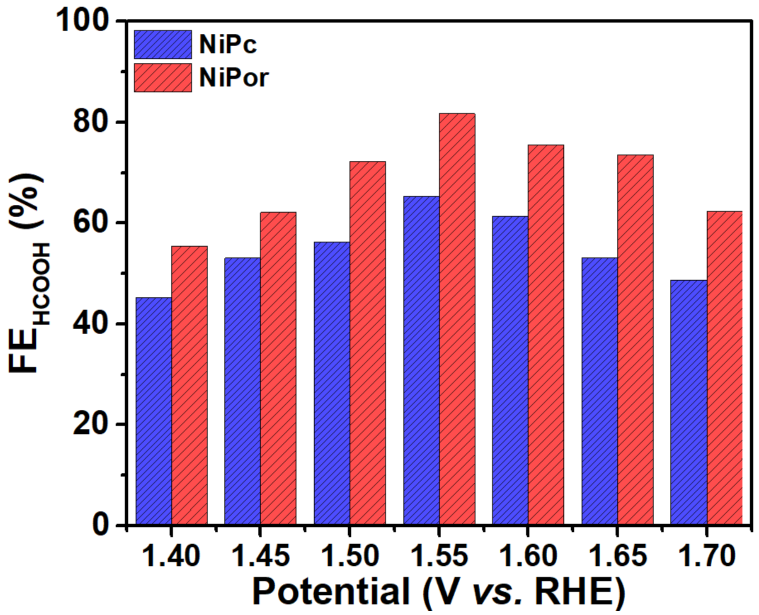

Figure S21. FE_{HCOOH} of NiPc and NiPor for MOR test.

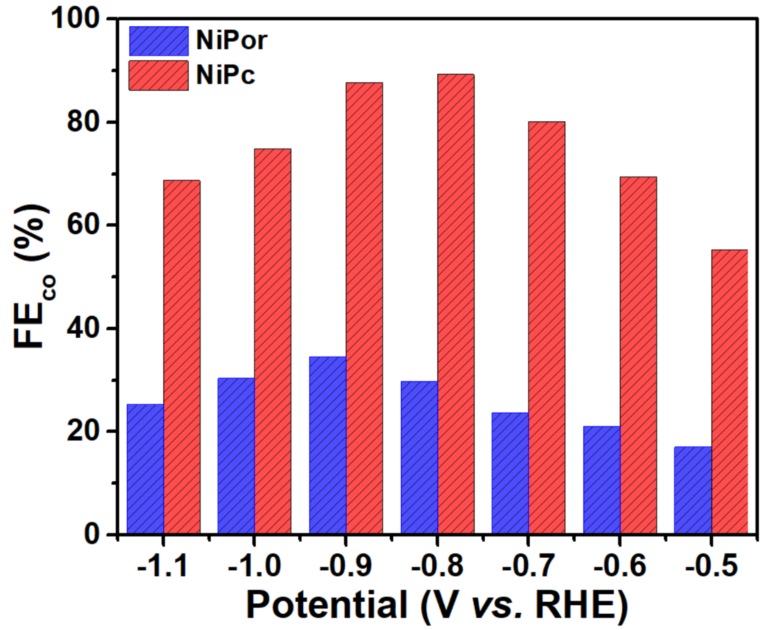

Figure S22. FE_{CO} of NiPc and NiPor for ECR test.

S2.3. Electrocatalytic ECR coupling MOR experiments

All electrochemical tests were applied in an air tight H-cell (Tianjin Aida Heng sheng Technology, China) with separated the cathodic and anodic chambers by Nafion 117 membrane. The standard two-electrode system, i.e. catalyst-modified carbon fiber papers both as working anode and cathode electrode, and the ECR coupling MOR tests on the electrochemical workstation (Bio-Logic VSP) and the CO_2 saturated 0.5 M $KHCO_3$ and 1 M CH_3OH in 1 M KOH were used as electrolyte. The potential range of 1.8 to 2.4 V (cell voltage, step size = 0.1 V) was applied during the ECR coupling MOR test and calculated the faradaic efficiency and current density. The yield of CO and H_2 was quantified by gas chromatography (GC-7920, CEAulight, China). The yield HCOOH was quantified by ion chromatography (Ion Chromatography System, Themorpher, China). The working electrode was similar to the preparation of ECR. The polarization curves results were obtained by performing linear sweep voltammetry (LSV) mode with a scan rate of 5 mV s^{-1}.

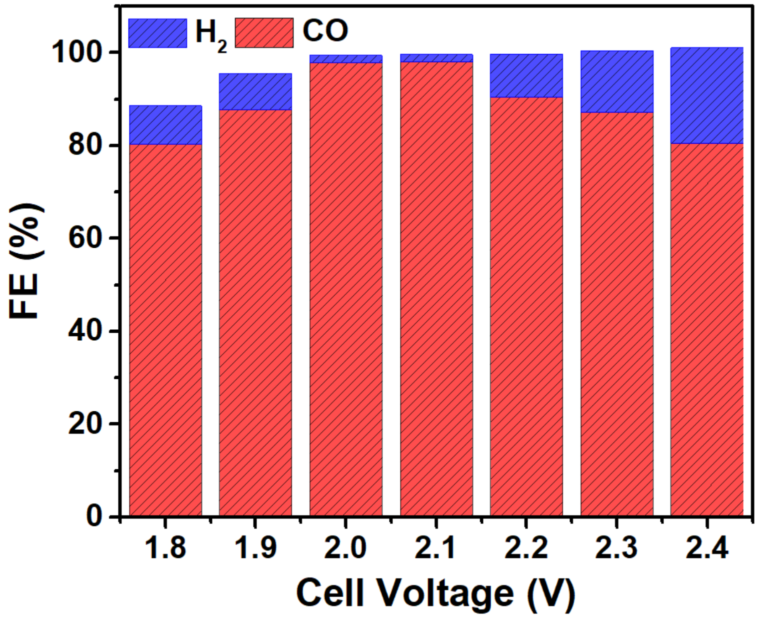

Figure S23. Faradaic efficiencies of ECR half reaction for the MOR || ECR cell using NiPc-NiPor COF as anode and cathode. Anode: 1 M KOH with the addition of 1 M methanol; Cathode: 0.5 M $KHCO_3$ solution saturated with CO_2.

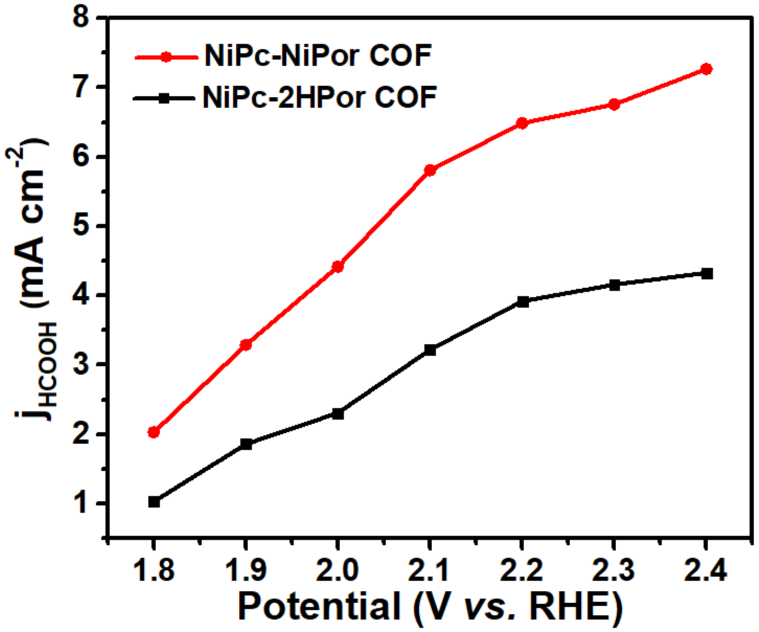

Figure S24. j_{HCOOH} of MOR half reaction for the MOR || ECR cell using NiPc-NiPor COF or NiPc-2HPor COF as anode and cathode.

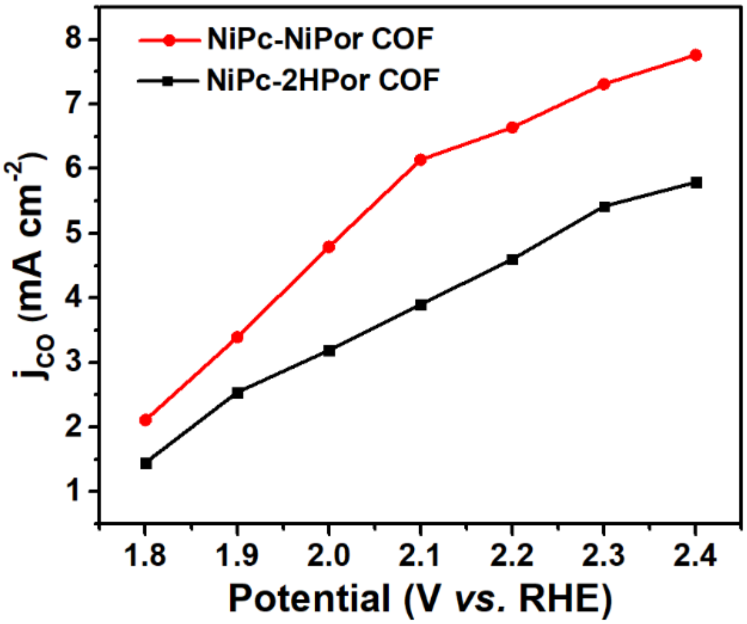

Figure S25. j_{CO} of ECR half reaction for the MOR || ECR cell using NiPc-NiPor COF or NiPc-2HPor COF as anode and cathode.

S2.4. Analysis of liquid products

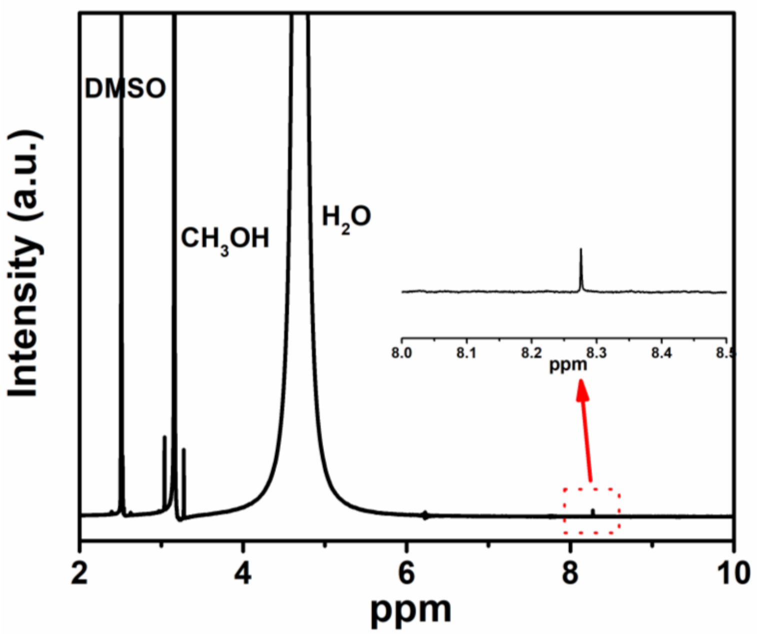

Figure S26. Characterization for the liquid product of NiPc-NiPor COF during MOR process in MOR || ECR cell by ^{1}H NMR.

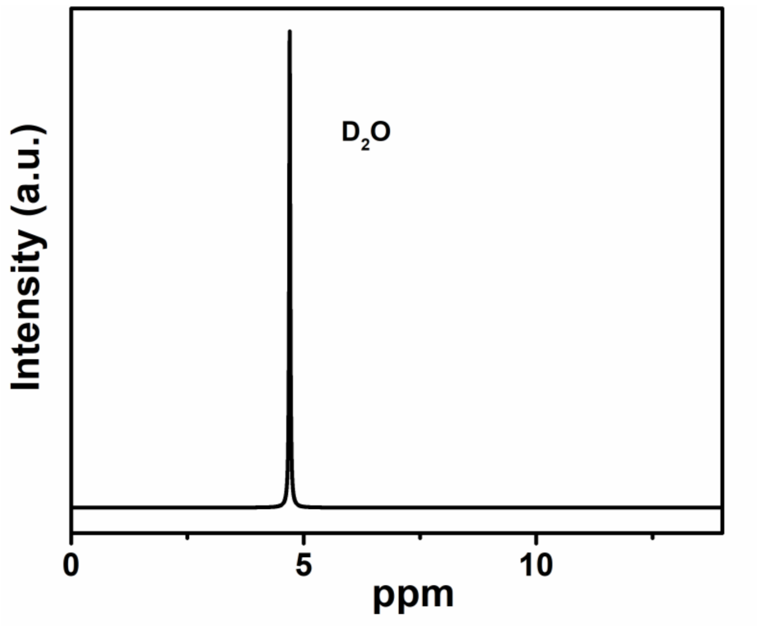

Figure S27. Characterization for the liquid product of NiPc-NiPor COF during ECR process in MOR || ECR cell using NiPc-NiPor COF as anode and cathode by ^{1}H NMR.

S2.5. Stability test

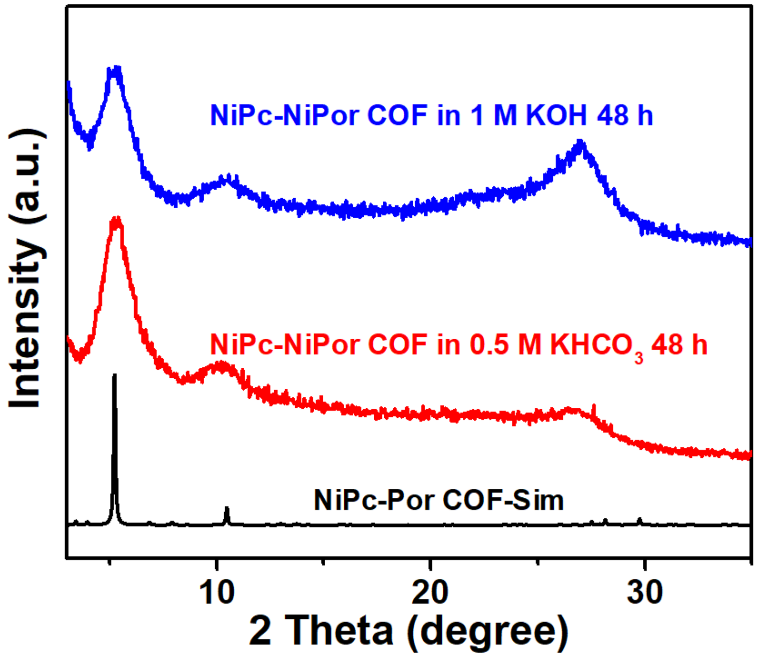

Figure S28. PXRD patterns of NiPc-NiPor COF immersed in 1 M KOH aqueous solution and 0.5 M $KHCO_3$ aqueous solution for 48 hours, respectively.

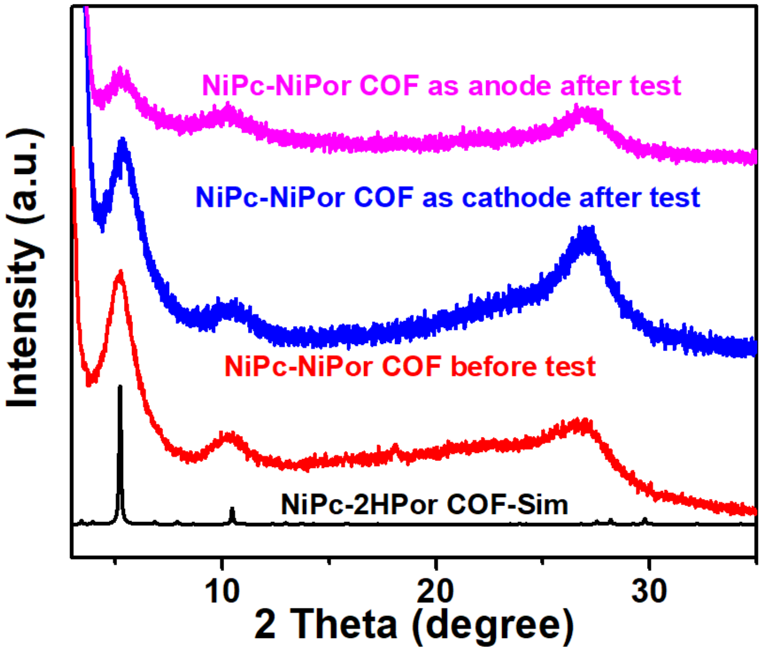

Figure S29. PXRD patterns of NiPc-NiPor COF before and after electrocatalytic reaction, which shows still maintaining crystallinity.

S3. Computational methods

S3.1. DFT calculations

All of the potential energy surface calculations were performed with at the (U)M06[3]/[6-31G(d)/LanL2DZ[4](Ni)] level. A pruned numerical integration grid (99,590) was used via the keyword Int = Ultrafine. Frequencies were calculated at the same theoretical level to verify the stationary points to be equilibriums or be transition states. A better basis set system was employed to evaluate the single-point energy at the the SMD(water)/ (U)M06-D3/[6-311++G(d, p)/SDD[5](Ni)] level, and combined it with the gas phase Gibbs free energy corrected by the thermodynamic energy (see below for details) to assess the free energy at 298.15 K and 1 atm in this work. All of the above calculations were performed with Gaussian 09[6] program.

We evaluated the electronic energy (E_{sol}) with zero-point energy correction in solution. For each species, the E_{sol} is defined through equation (S1):

$$G_{sol} = E_{sol}^{pot} + E_{gas}^{v_0} \qquad \text{(S1)}$$

where E_{sol}^{pot} is the potential energy including non-electrostatic energy in solution and $E_{gas}^{v_0}$ represents the zero-point vibrational energy in the gas phase. In a bimolecular process, such as the coordination of the CO_2 with the Ni complex, the entropy change must be taken into consideration because the entropy considerably decreases. In this case, Gibbs energy (G_{sol}^{o}) must be evaluated as follows:

$$G_{sol}^{o} = H_0 - T(S_r^o + S_v^o + S_t^o)$$
$$= E^T + P\Delta V - T(S_r^o + S_v^o + S_t^o)$$
$$= E_{sol} + E_{therm} - T(S_r^o + S_v^o + S_t^o) \qquad \text{(S2)}$$

where ΔV is 0 in solution, E_{therm} is the thermal correction by translational, vibrational, and rotational movement, and S_r^o, S_v^o, and S_t^o are rotational, vibrational, and translational entropies, respectively. In general, the Sackur-Tetrode equation is used to evaluate translational entropyS_t^o. In solution, however, the usual Sackur-Tetrode equation cannot be directly applied to the evaluation of S_t^o, because the translation movement is suppressed very much in solution[7]. In this context, the translational entropy was corrected with the method developed by Whitesides et al.[8], where the rotational entropy was evaluated in a normal manner. Thermal correction and entropy contributions of vibration movements to the Gibbs energy were evaluated with the frequencies calculated at 298.15 K and 1 atm.

S3.2. Structural modeling

Structural modeling of NiPc-2HPor COF was carried out in the BIOVIA Materials Studio 2019 software package. The theoretical models were optimized by the Forcite module. The Pawley refinement of the experimental PXRD were conducted to optimize the lattice parameters iteratively, in which a Pseudo-Voigt profile function was used for the profile fitting (peak asymmetry, peak broadening, and zero shift error were considered) until the R_{WP} and R_P value converges. Unit cell dimension was set to the theoretical parameters.

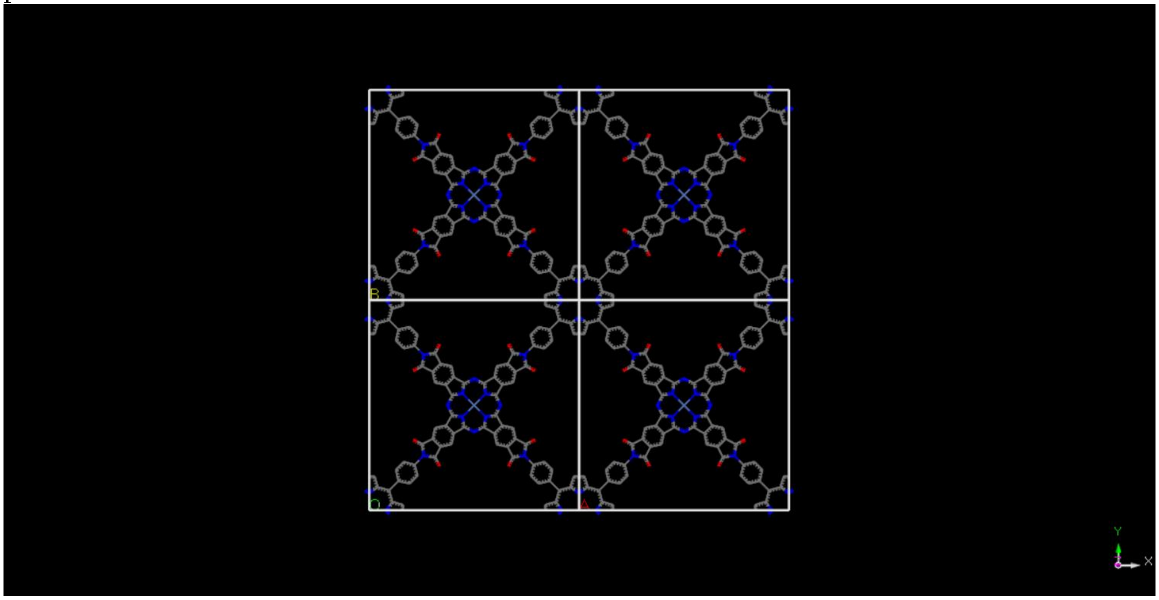

Figure S30. The unit-cell structures (2×2×2) of NiPc-2HPor COF deriving from the AA stacking model.

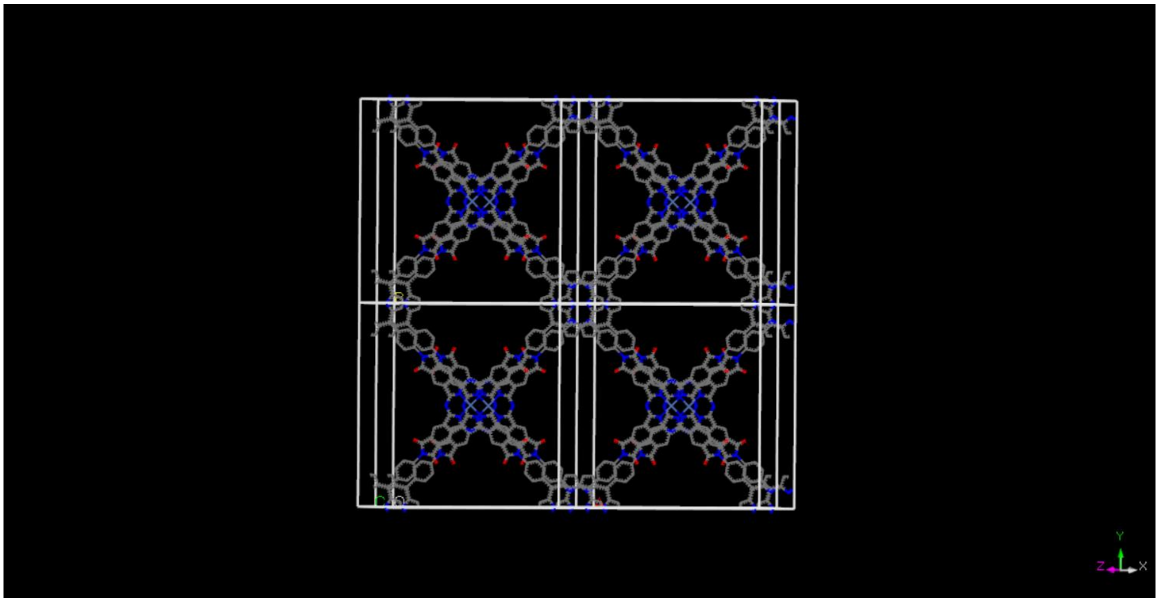

Figure S31. The unit-cell structures (2×2×2) of NiPc-2HPor COF deriving from the AA slipped stacking model.

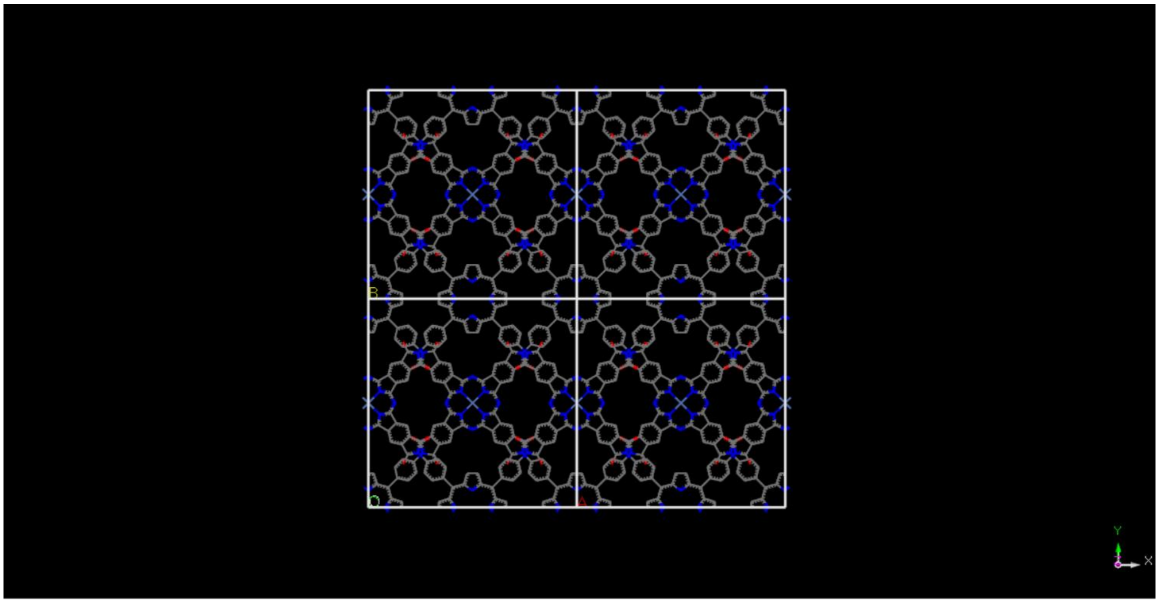

Figure S32. The unit-cell structures (2×2×2) of NiPc-2HPor COF deriving from the AB stacking model.

Table S2. Fractional atomic coordinates for the unit cell of NiPc-2HPor COF with AA slipped stacking.

AA slipped stacking	Space group: PM (6)			
	Lattice type: monoclinic			
	a（Å）= 25.7859			
	b（Å）= 25.7859			
	c（Å）= 3.4637			
	**α = γ = 90°，β= 120°**			
	**Cell volume:** 824.753			
Label	Atom	x	y	z
C1	C	0.16824	0.97384	2.48082
C2	C	0.11301	0.95786	2.40405
C3	C	0.23693	0.16644	2.42857
C4	C	0.1986	0.1275	2.44368
O5	O	0.79683	0.33215	2.46802
C6	C	0.65888	0.30143	2.29979
C7	C	0.60339	0.31474	2.27043
C8	C	0.59386	0.36812	2.32259
C9	C	0.5454	0.39454	2.31057

C10	C	0.6826	0.25396	2.25817
C11	C	0.82865	0.02616	2.12842
C12	C	0.88388	0.04214	2.20515
C13	C	0.75997	0.83356	2.18065
C14	C	0.79829	0.8725	2.16554
O15	O	0.20006	0.66785	2.14123
C16	C	0.33801	0.69857	2.30945
C17	C	0.3935	0.68526	2.33881
C18	C	0.40304	0.63188	2.28664
C19	C	0.45149	0.60546	2.29865
C20	C	0.31429	0.74604	2.35105
C21	C	0.02527	0.15909	2.31603
C22	C	0.04172	0.10828	2.3217
C23	C	0.83674	0.22939	2.37406
C24	C	0.87439	0.1902	2.35546
O25	O	0.65617	0.7873	2.17867
C26	C	0.70182	0.66156	2.38205
C27	C	0.69161	0.60793	2.42271
C28	C	0.63544	0.59446	2.38269
C29	C	0.60909	0.54656	2.38261
C30	C	0.75033	0.68844	2.3968
C31	C	0.97162	0.84091	2.29321
C32	C	0.95517	0.89172	2.28753
C33	C	0.16015	0.77061	2.23517
C34	C	0.12251	0.8098	2.25377
O35	O	0.34073	0.2127	2.4306
C36	C	0.29507	0.33844	2.22718
C37	C	0.30528	0.39207	2.18652
C38	C	0.36145	0.40554	2.22654
C39	C	0.38781	0.45344	2.22661
C40	C	0.24656	0.31156	2.21243
N41	N	-0.00155	0.075	2.30461
C42	C	0.09801	0.09579	2.36056
C43	C	0.13923	0.13713	2.35647
C44	C	0.21858	0.21863	2.31948
C45	C	0.89888	0.90421	2.24865
C46	C	0.85766	0.86287	2.25275
C47	C	0.77832	0.78137	2.28975
N48	N	0.73909	0.74063	2.31586
N49	N	0.25781	0.25937	2.29337
N50	N	0.55332	0.44719	2.33442
N51	N	0.44357	0.55281	2.27481
N52	N	0.49845	0.62965	2.30461
N53	N	0.07756	0	2.36847
N54	N	0.91933	0	2.24071

N56	N	0.63478	0.5	2.4064
N57	N	0.36211	0.5	2.20282
Ni58	Ni	0.49844	0.5	2.30453

References

1. Lu M, Liu J and Li Q *et al.* Rational design of crystalline covalent organic frameworks for efficient CO_2 photoreduction with H_2O. *Angew Chem Int Ed* 2019; **58**: 12392-97.

2. Sun S-N, Dong L-Z and Li J-R *et al.* Redox-active crystalline coordination catalyst for hybrid electrocatalytic methanol oxidation and CO_2 reduction. *Angew Chem Int Ed* 2022; **61**: e202207282.

3. Zhao Y and Truhlar D G. The $M0_6$ suite of density functionals for main group thermochemistry, thermochemical kinetics, noncovalent interactions, excited states, and transition elements: two new functionals and systematic testing of four $M0_6$-class functionals and 12 other functionals. *Theor Chem Acc* 2008; **120**: 215-41.

4. Hay P J and Wadt W R. Ab Initio effective core potentials for molecular calculations. potentials for K to Au including the outermost core orbitals. *The Journal of Chemical Physics* 1985; **82**: 299-310.

5. Dolg M, Wedig U and Stoll H *et al.* Energy‐adjusted ab initio pseudopotentials for the first row transition elements. *The Journal of Chemical Physics* 1987; **86**: 866-72.

6. Frisch M J, G W T T and Schlegel H B *et al.* Fox, Gaussian 09 Revision D.01, Gaussian Inc., Wallingford CT. 2013.

7. Sakaki S, Ohnishi Y-Y and Sato H Theoretical and computational studies of organometallic reactions: successful or not? *The Chemical Record* 2010, 10 (1), 29-45.

8. Mammen M, Shakhnovich E I and Deutch J M *et al.* Estimating the entropic cost of self-assembly of multiparticle hydrogen-bonded aggregates based on the cyanuric acid·melamine lattice. *The Journal of Organic Chemistry* 1998; **63**: 3821-30.
